# Modulation of the intestinal microbiota of broilers supplemented with monensin or functional oils in response to challenge by *Eimeria* spp.

**DOI:** 10.1371/journal.pone.0237118

**Published:** 2020-08-07

**Authors:** Alexandre Maciel Vieira, Tatiany Aparecida Teixeira Soratto, Kátia Maria Cardinal, Glauber Wagner, Lucélia Hauptli, André Luis Ferreira Lima, Fabiano Dahlke, Diego Peres Netto, Priscila de Oliveira Moraes, Andréa Machado Leal Ribeiro

**Affiliations:** 1 Department of Animal Science and Rural Development, Universidade Federal de Santa Catarina, Florianópolis, Santa Catarina, Brazil; 2 Laboratory of Bioinformatics, Center of Biological Sciences, Universidade Federal de Santa Catarina, Florianópolis, Santa Catarina, Brazil; 3 Department of Animal Science, Laboratory of Animal Science, Universidade Federal do Rio Grande do Sul, Porto Alegre, Rio Grande do Sul, Brazil; USDA-Agricultural Research Service, UNITED STATES

## Abstract

The objective of this study was to evaluate the effect of supplementation with 100ppm sodium monensin or 0.15% of a blend of functional oils (cashew nut oil + castor oil) on the intestinal microbiota of broilers challenged with three different *Eimeria* spp. The challenge was accomplished by inoculating broiler chicks with sporulated oocysts of *Eimeria tenella*, *Eimeria acervulina*, and *Eimeria maxima* via oral gavage. A total of 864, day-old male broiler chicks (Cobb) were randomly assigned to six treatments (eight pens/treatment; 18 broilers/pen) in a 3 × 2 factorial arrangement, composed of three additives (control, monensin or blend), with or without *Eimeria* challenge. Intestinal contents was collected at 28 days of age for microbiota analysis by sequencing 16s rRNA in V3 and V4 regions using the Illumina MiSeq platform. Taxonomy was assigned through the SILVA database version 132, using the QIIME 2 software version 2019.1. No treatment effects (p > 0.05) were observed in the microbial richness at the family level estimated by Chao1 and the biodiversity assessed by Simpson’s index, except for Shannon's index (p < 0.05). The intestinal microbiota was dominated by members of the order Clostridiales and Lactobacillales, followed by the families Ruminococcaceae, Bacteroidaceae, and Lactobacillaceae, regardless of treatment. When the controls were compared, in the challenged control group there was an increase in Erysipelotrichaceae, Lactobacillaceae, Bacteroidaceae, Streptococcaceae, and Peptostreptococcaceae, and a decrease in Ruminococcaceae. Similar results were found for a challenged group that received monensin, while the blend partially mitigated this variation. Therefore, the blend alleviated the impact of coccidiosis challenge on the microbiome of broilers compared to monensin.

## Introduction

The more balanced the microbiome, the more resilient the intestine becomes when challenged [1,2]. *Eimeria* spp. parasites, which are responsible for coccidiosis, a disease that seriously affects the global poultry industry, are one of the factors that cause changes in intestinal microbiota Coccidiosis causes damage to the intestinal epithelium, changes in the immune response of broiler chickens, reduces the digestion of nutrients, and consequently reduces performance indices [3–5]. Coccidiosis has also been shown to affect the diversity and composition of the microbial community [4]. For example, increases in the leakage of plasma proteins into the lumen caused by the parasite may serve as a substrate for the proliferation of *Clostridium perfringens*. Although this bacterium is part of the normal microbiota of the cecum of broilers, when there is a significant increase in its proliferation in the small intestine it can cause necrotic enteritis [3,4].

Ionophores have been the main choice of drug for controlling coccidiosis. Monensin is an ionophore that forms fat-soluble complexes with sodium and potassium, causing increased permeability of the coccidial membrane, which leads to toxic effects and death due to depletion of cellular energy [6]. This mechanism also occurs in bacteria and may explain some of the changes seen in the intestinal microbiota, although there are limited studies that relate the use of monensin to its impact on the microbiota. Lu et al. [7] observed that monensin supplementation produced a greater relative abundance of gram-negative bacteria, such as the Proteobacteria phylum, Clostridia class, and Bacteroides genera. Danzeisen et al. [8] observed that the combination of monensin with virginiamycin and tylosin increased the presence of *E*. *coli*, *Lactobacillus* spp., and *Enterococcus* spp. However, the continuous use of monensin can cause parasite resistance [9], which has stimulated research into alternative additives with similar potentials, such as phytogenics [10–13].

Phytogenic feed additives can have antimicrobial and anti-inflammatory properties, because they are composed of a complex mixture of volatile substances, such as terpene hydrocarbons, simple alcohols, and aldehydes, amongst other pharmacologically active compounds [14]. Of the phytogenic additives, functional oils are defined as those that have an action beyond the nutritional value [15]. The commercial blend containing functional oils of cashew nut shell liquid and castor oil has demonstrated the positive effects in growth performance, antimicrobial and anticoccidial activity, with a reduction in the severity of cecal lesions in chickens infected with *Eimeria* spp. [15].

The objective of this study was to evaluate the effects of supplementation with either monensin or blend functional oils on the intestinal microbiota of broilers challenged with coccidiosis.

## Material and methods

### Ethics statement

The work described here was conducted under protocol number 29814 approved by the Ethics Committee on Animal Use of the Federal University of Rio Grande do Sul, following the legislation for the protection of animals used for scientific purposes (NIH Publications No. 8023, revised 1978). Birds were monitored twice a day, systematically observed by trained people who evaluated possible coccidiosis clinical signs, like: occurrence of mucus-like or bloody diarrhea; dehydration diagnosed by responses of wrinkled skin, dull eyes; decreased appetite detected by decrease in feed intake; occurrence of ruffled feathers; listlessness, detected by activity lack of the bird and; stunted growth, visually lower than the group average. In case of early evidence associated with possible death risk or specific signs of severe suffering or distress, the birds could have euthanized. But these specific situations (euthanasia) were not necessary in this study.

### Bird husbandry and experimental design

A total of 864, day-old male broiler chicks (Cobb 500) were obtained from a commercial hatchery and housed in two identical experimental facilities with a controlled-temperature room, one for the challenged, and one for the unchallenged birds, thus avoiding cross-contamination. The rooms were composed of 48 pens with an initial density of 18 birds per pen. Each group was housed in a 1 m^2^ pen equipped with two nipple drinkers and one tubular feeder. Food and water were provided *ad libitum* throughout the 28 days of the experimental period. The nutritional program consisted of three phases: pre-starter (1 to 7 d), starter (8 to 21 d), and grower (22 to 28 d), formulated to provide the nutritional requirements recommended by the Brazilian Tables of Poultry and Swine [16].

The experimental design was completely randomized in a 3 × 2 factorial arrangement, composed of feed additives (basal diet, 100 ppm sodium monensin, or 0.15% blend) and sanitary challenge (challenged or unchallenged with coccidiosis). Both feed additives, the oil blend (Oligo Basics Agroind. Ltd, Cascavel, PR, Brazil) and monensin sodium (Elanco Animal Health, Greenfield, IN, USA), were included by replacing an inert ingredient (kaolin) in the basal diet for all phases.

### Challenge and sample collection

At 14 days of age, 1 mL of a saline solution containing sporulated oocysts of *E*. *tenella* (10 × 10^3^ oocysts), *E*. *acervulina* (200 × 10^3^ oocysts), and *E*. *maxima* (80 × 10^3^ oocysts) were inoculated by oral gavage. The oocysts were acquired from the Laboratório de Biologia Molecular de Coccídias (University of São Paulo, Brazil). To similarly stress all treatments, unchallenged broilers received 1 mL of saline *per os*.

At 28 days of age, a homogenized sample of the small intestinal digesta from three broiler chickens per replicate (three replicates) was obtained, totaling 18 samples. A 10 cm portion of each segment: duodenum segment (from the pylorus exit to the end of the descending duodenal loop), jejunum (descending duodenal loop to the Meckel's diverticulum), and ileum (diverticulum to ileocecal insertion) was homogenized, pooled, and immediately stored at -20°C until further analysis.

### DNA extraction, PCR amplification, and sequencing

The small intestine samples were placed in a 1.5 mL sterile tube and sent to Neoprospecta Microbiome Technologies (Florianópolis-SC, Brazil). All procedures were performed according to the method previously described [17]. Sample preparation and sequencing were performed by Neoprospecta Microbiome Technologies. For total DNA extraction, the commercial QIAamp DNA Stool Mini Kit (QIAGEN, Hilden, Germany) was used according to the manufacturer’s instructions. Samples DNA quality were measured using a Nanodrop ND-1000 spectrophotometer (ND-1000; Thermo Scientific, Waltham, MA, USA), by measuring the absorbance values at 260 and 280 nm. The V3 and V4 regions of the 16S rRNA genes were PCR-amplified using the 341F (5′-CCTACGGGRSGCAGCAG-3′) and 806R (5′-GGACTACHVGGGTWTCTAAT-3′) primers, with Illumina adapters (Life Technologies, Carlsbad, CA, USA). The amplification was performed in 35 cycles at an annealing temperature of 50°C. Sequencing was performed by Illumina MiSeq (Illumina, San Diego, CA, USA) using V2x300 kits, with a single-end 300 nt run.

### Sequence analysis

Read quality was evaluated using FastQC software (version 0.11.5), and poor quality reads and adapters were removed (quality lower than 30). All of the following steps were implemented using QIIME 2 (version 2019.1) software [18]. The reads were subjected to a Denoising approach for low-quality sequence removal, sequencing error correction, chimera removal, and identification of the variations of sequence amplification (ASVs) employing the DADA2 methodology with default parameters, and 260 truncated read length. ASVs below a frequency of 0.1% in the samples were removed. Taxonomy was attributed to ASVs using the SILVA database (version 132), with a 97% correspondence.

### Statistical analysis

Relative abundance, alpha rarefaction, alpha (Chao-1, Shannon, and Simpson), and beta diversity indices were determined using the program R, version 3.6 (https://www.R-project.org/), and plyr (v. 1.8.4) [19], reshape2 (v. 1.4.3) [20], and phyloseq (v. 1.14.0) [21] packages. Beta diversity was estimated after sequence number normalization by randomly choosing sequences in the samples so that each sample obtained the same number of sequences. After normalization, a principal coordinate analysis (PCoA) was performed and a heatmap was obtained using the Bray-Curtis dissimilarity index with the vegan (v. 2.4.1) [22] and heatmaps (v. 1.8.0) [23] packages. A Venn diagram was generated using the Venn (v. 1.7) [24] R package. Relative abundance and alpha diversity were tested using the Kruskal–Wallis test. Similarity analysis (ANOSIM) tests were conducted based on beta diversity.

## Results

### Sequencing data

A total of 728,462 quality trimmed sequences were produced with an average number of sequences per sample of 40,470.11 ± 7,580.40 (S1 Table). The reads were processed and classified into 1,088 variations in sequence amplification (ASVs).

Rarefaction curves generated from ASVs (S1 Fig) showed high sequencing coverage in all samples. The rarefaction curves tended to reach the saturation plateau, showing that the microbiota of the 18 samples was deep enough to estimate phenotype richness and microbial community diversity.

#### Variation in alpha and beta diversity of microbiota

**Fig 1:**
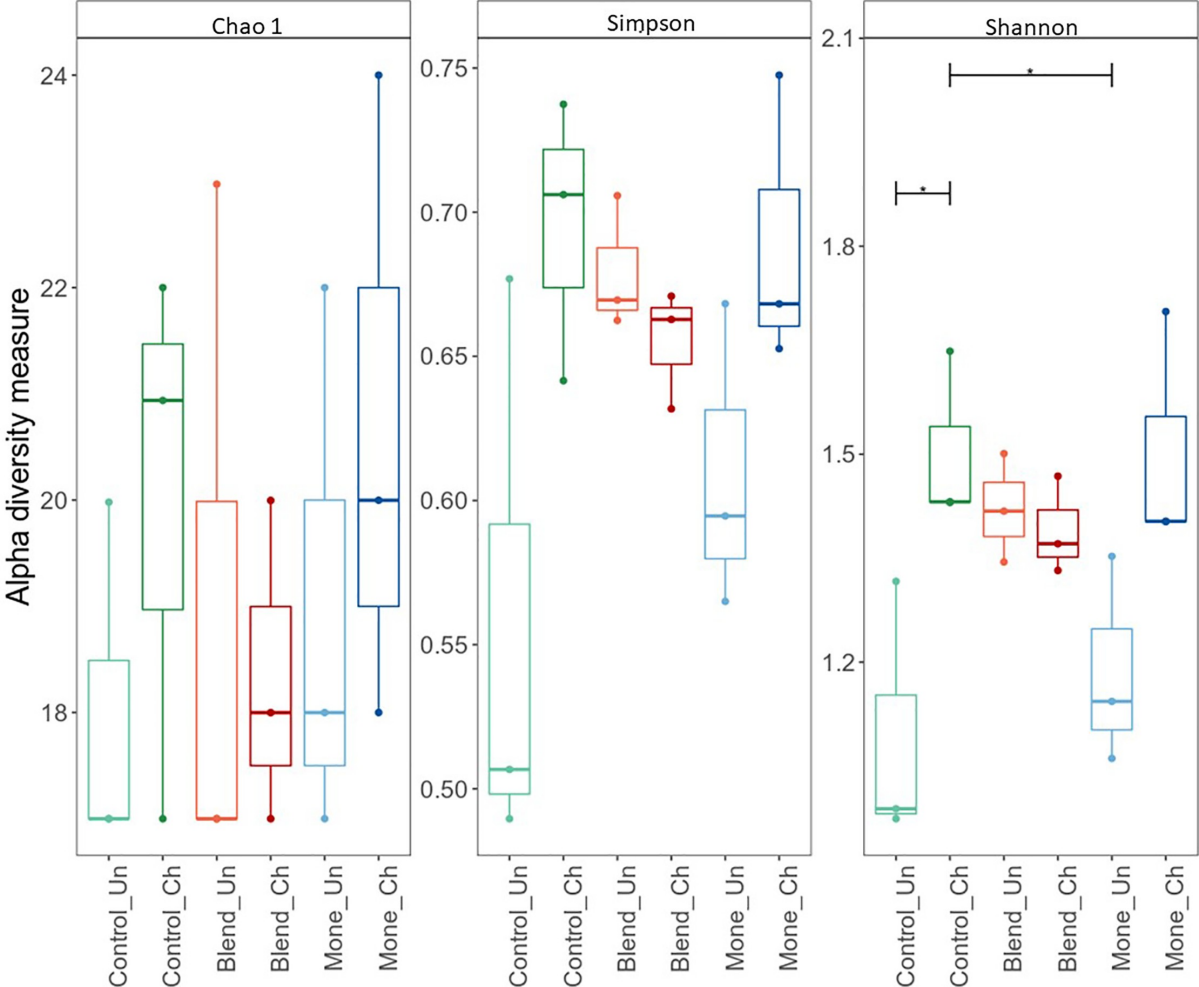
Alpha diversity at the family level in the small intestinal digesta of broiler chickens at 28 days of age. Experiment consisted of three feeding treatments: basal diet (control), sodium monensin (Mone), or oil blend, with (CH) or without (UN) a coccidiosis sanitary challenge.

The Chao 1 index (minimum number of ASVs present in a sample) was based on the richness of families present in the sample. The Shannon index considered uniformity in family abundance, and the Simpson index was based on dominance of abundance (Fig 1).

However, only the Shannon index showed significant differences between the six groups after infection (p <0.05). While the Shannon index increased in the monensin and challenged control groups, it did not change in the blend group (S2 Table).

**Fig 2:**
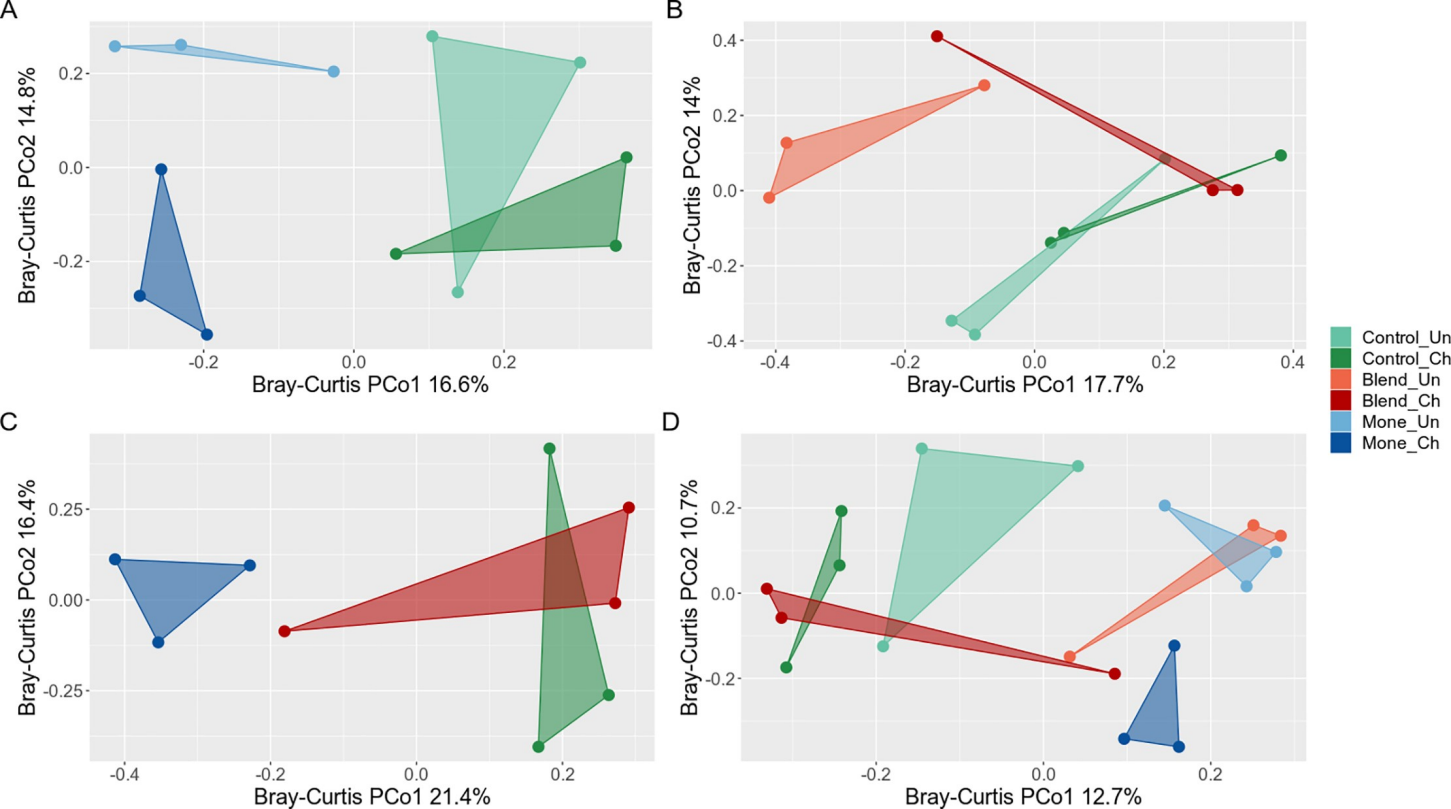
Principal coordinate analysis (PCoA) of beta diversity based on Bray-Curtis dissimilarity in a broiler experiment composed of three feeding additives, basal diet (control), sodium monensin (Mone), or blend (Blend) and coccidiosis challenge (CH) or unchallenged (UN). (A) Comparison between Mone and control treatments (Adonis with 999 permutations, p = 0.002). (B) Comparison between blend and bcontrol treatments (Adonis with 999 permutations, p = 0.252). (C) Comparison between Mone, blend, and control treatments challenged with coccidiosis (Adonis with 999 permutations, p = 0.179). (D) Between all treatments (Adonis with 999 permutations, p = 0.002).

To analyze the inter-individual differences, the Bray-Curtis dissimilarity index (CB) was used. which showed that the treatments had a different microbial compositions (Adonis with 999 permutations, p = 0.002). Despite the lack of homogeneity in the dispersion, the PCoA graph (Fig 2) based on the Bray-Curtis dissimilarity matrix showed that the microbial populations of the challenged animals that received the additives were presented in closer groups than in the control treatment.

#### Common and unique microbial populations

**Fig 3:**
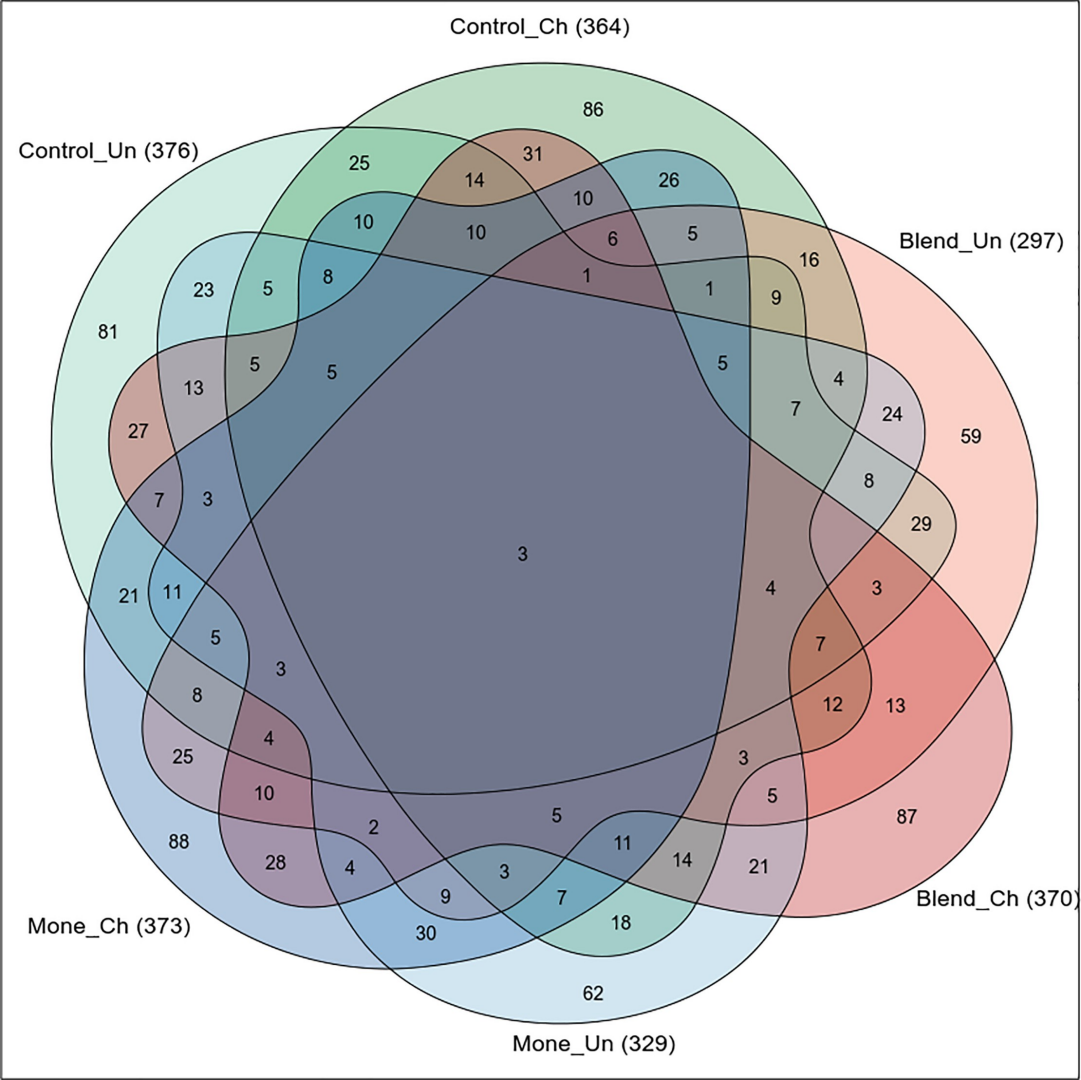
Common and unique variations in sequence amplification (ASVs) in broilers receiving three feed additives, basal diet (control), sodium monensin (Mone), or blend and challenged (CH) or not challenged (UN) with coccidiosis. The Venn diagram shows the numbers of ASVs that are shared or not shared by the six treatments, depending on the overlaps.

A paired comparison of the microbial similarity between treatments as well as an analysis of common ASVs, shown in the Venn diagram, was conducted to investigate the microbial community. A total of three ASVs were common to all groups, the number of ASVs present in only one group ranged from 59 for the unchallenged blend to 88 for challenged monensin. Challenging with coccidiosis increased the number of ASVs present only in one group, going from 86 to 88 for Ch, and from 59 to 81 for the blend, monensin, and control treatments, respectively (Fig 3) for Un.

**Fig 4:**
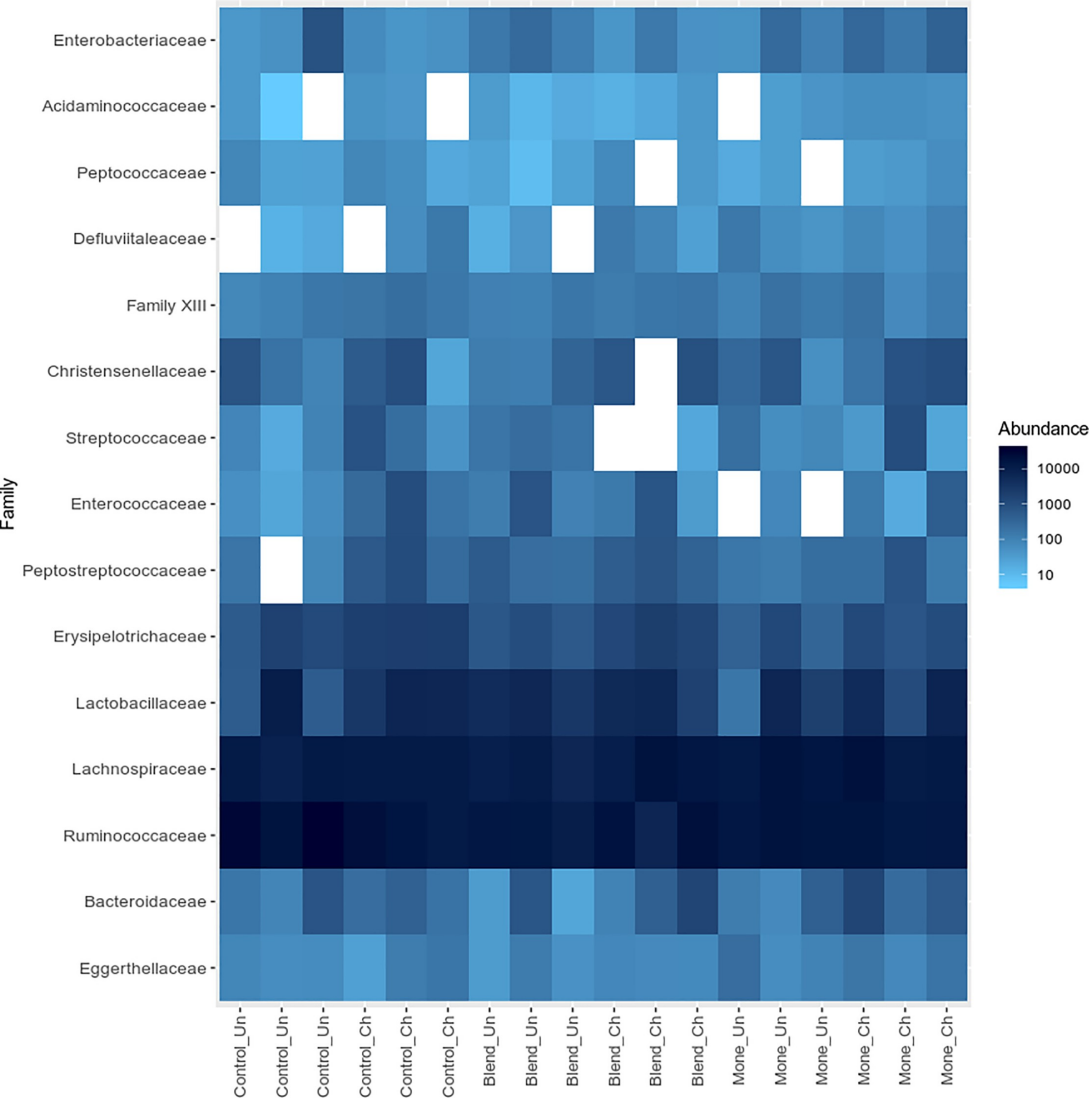
Microbial distribution of the eighteen samples. Rows represent the 15 predominant bacterial families ordinated by Bray Curtis distance and PCoA metric. Columns represent the eighteen samples, and the values in the heatmap represent the abundance log_10_ transformed of each bacterial family, indicated on the right side of the figure.

**Fig 5:**
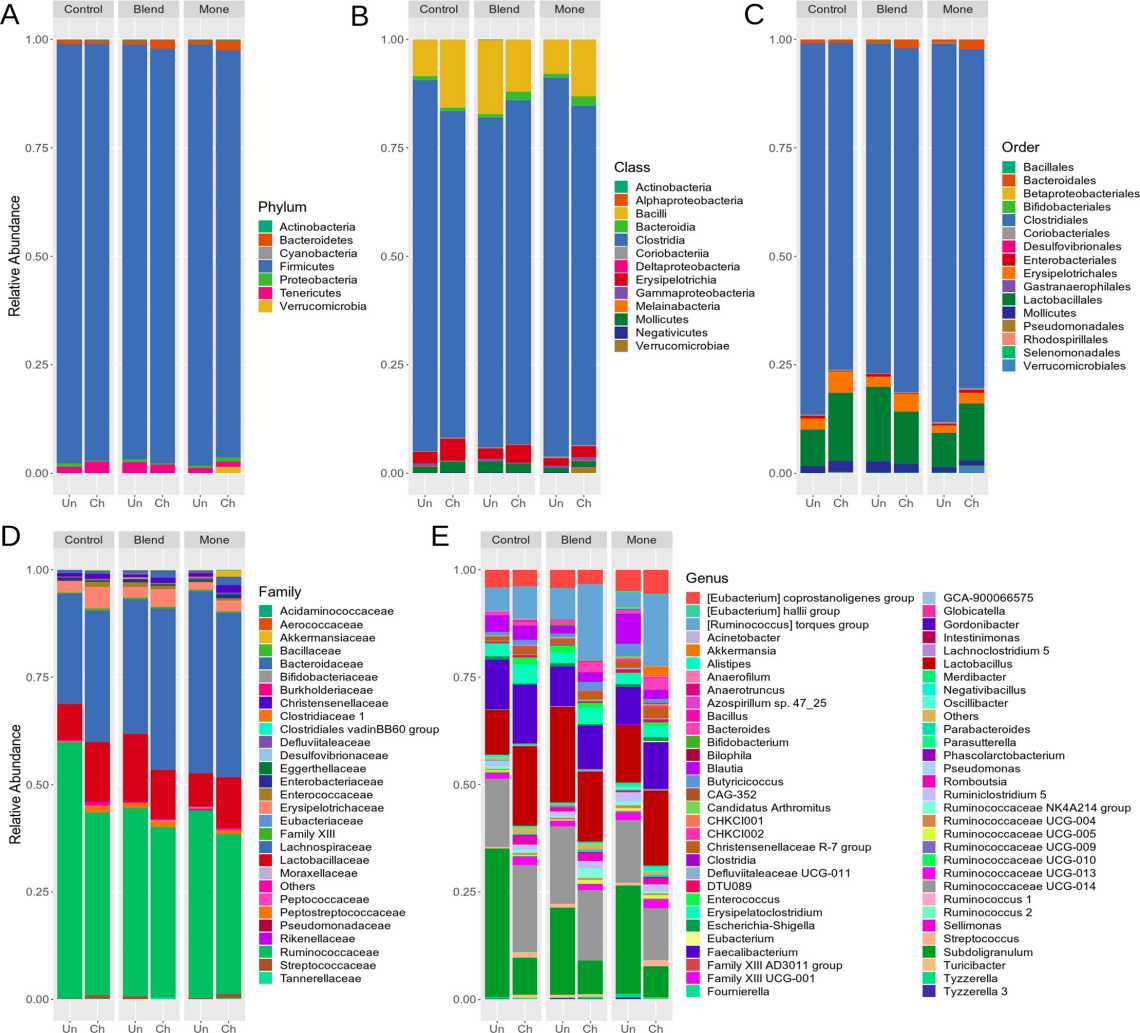
The relative abundance of gut microbiota in 28 day old broilers receiving three feed treatments; basal diet (control), sodium monensin (Mone), or oil blend (Blend), with (CH) and without (UN) a coccidiosis challenge. Relative abundance is presented as a percentage (%) of bacteria at (A) phylum, (B) class, (C) order, (D) family, and (E) genus level. Minor bacterial genera and unassigned values were included as “others”.

The dominant families in the samples were Ruminococcaceae and Lachnospiraceae, followed by Lactobacillaceae, Bacteroidaceae, and Erysipelotrichaceae (Fig 4). A complete list of the sequences identified (relative abundance) by treatment and challenge is provided in S3 Table. All sequences were classified into seven phyla, although three phyla were more common (> 1%): Firmicutes, Bacteroidetes, and Tenericutes (Fig 5A and S3 Table). Firmicutes was the most abundant phylum in all treatments, regardless of the challenge (> 94%). However, birds that received monensin and were challenged showed a reduction in this phylum (97.05% vs. 94.07%), with more Bacteroidetes and Verrumicrobia and less Tenericutes.

At the class level, Clostridia was the most abundant class upper to 75% of the sequences of the 18 samples, regardless of the additive. The challenged broilers showed lower levels of this class, except for those supplemented with blend, which showed difference between challenged and unchallenged broilers (Fig 5B and S3 Table). At the opposite the abundance of Bacilli class there has increased in the broilers that were challenged, except the group treated with the blend.

An increase in Bacteroidia (~ 0.80% vs. ~ 2.05%) was observed for the blend and monensin challenged groups. There was an increase in the abundance of Erisypelotrichia and Mollicutes, regardless of the treatment, when broilers were challenged, with the exception of the treatment receiving the blend, in which the Mollicutes class was reduced. The class Melainabacteria was identified in low abundance (<0.1%) only in the challenged birds.

Thirty families were identified, of which seven had a relative abundance > 1% (Fig 5D and S3 Table). The Ruminococcaceae family was predominant in all groups (> 31%), mainly in non-challenged animals, and lower percentages were found in the blend and monensin groups. The Lachnospiraceaefamily was the second most abundant (> 25%), and blend or monensin challenged broilers showed similar abundance (~ 37%). Lactobacillaceae showed higher indeces in challenged broilers (> 11%), except for the treatment with the blend (15.06% vs. 11.64%, unchallenged, and challenged, respectively). The abundance of the Erysipelotrichaceae family was increased in challenged broilers. A higher percentage of Bacteroidaceae were observed in the monensin and blend groups (> 1.5%) when broilers were challenged. Streptococcaceae significantly increased in challenged broilers supplemented with monensin (1.16%). Conversely, Streptococcaceae was visibly reduced when blend was used (∼0.02%).

## Discussion

The commercial blend of cashew nut liquid and castor oil has been shown to modulate the inflammatory response against *Eimeria* spp. In the absence of parasites, there was no stimulation of genes involved in the inflammatory response, demonstrating the blend was an effective tool to specifically modulate the immune system of birds afflicted with coccidiosis [25]. Monensin and blend effectively minimized the impact of coccidiosis at different times. While monensin acts as an antimicrobial, the blend modulates the intestinal microbiota with antimicrobial action against gram-positive bacteria, mainly *C*. *perfringens* and *S*. *aureus* [26]. In general, the blend improved the performance of the challenged birds after two weeks, resulting in a similar performance to the ionophore monensin. It seems that while monensin acts directly against the parasite, the blend acts as a modulator of the immune system and intestinal microbiota of birds.

Intestinal health is directly related to the profile of the microbiota that interacts with the host. The microbiota regulate absorption efficiency, antagonize the effects of pathogenic bacteria, improve intestinal integrity, and modulate immunity [27,28]. A coccidiosis challenge leads to changes in nutrient absorption and digestibility, with increased mucogenesis and membrane permeability, increasing the availability of nutrients, and the proliferation of pathogenic bacteria [1,28,29].

In this study, the coccidiosis challenge increased the microbial biodiversity, contrary to what was reported by Zhou et al. [5] and Bortoluzzi et al. [30]. However, the blend modulated this change, maintaining a narrower index between challenged and unchallenged animals, compared in the control and monensin treatments. The blend maintained the bacterial domain (total number of bacteria) after the challenge when analyzed via RT-PCR, while monensin reduced it [26].

In humans, weight loss is related to an imbalance in the Firmicutes: Bacteroidetes ratio in the intestinal microbiota, with a decrease in the Firmicutes and an increase in the Bacteroidetes, while the opposite was observed in obese rats [31]. The Firmicutes phylum is predominant in the gut poultry and has been linked to the efficiency of energy harvesting in various animals [8,32,33]. In this study, a decrease in the frequency of Firmicutes and an increase in Bacteroidetes was observed in challenged birds. In situations of dysbiosis, such as those caused by coccidiosis, some genera of Bacteroidetes can proliferate and become pathogenic, and consequently reduce the feeding efficiency of birds [34]. In this study, a lower ratio of Firmicutes: Bacteroidetes in monensin and the control was observed when compared to the blend, a fact that may explain the better improved performance of these birds [26].

The proliferation of *Lactobacillus spp*. was stimulated by the *Eimeria spp*. challenge which is in agreement with the results of Kley et al. [35], M'sadeq et al. [36], and Stanley et al. [3]. Several species of *Lactobacillus* have been associated with beneficial properties to the host, positively contributing to broiler weight gain, reducing injury scores, inhibiting cell invasion, and increasing mucosal integrity [37–39]. The increase in this family in the current study may be related to the triggering of an immunomodulatory response by *Lactobacillus* spp. facing the challenge. It has been demonstrated that many species of Lactobacilli act on the innate and acquired system stimulating immune cells to release pro-inflammatory cytokines such as tumor necrosis factor-alpha (TNF-α), interferon-gamma (IFN-γ), and interleukin-12 (IL-12) [40–44].

The Aerococcaceae family, another member of the phylum Firmicutes, was observed only in the group supplemented with the blend (challenged or not). This family is one of the bacteria that produce lactic acid [45,46], reducing the pH of the gastrointestinal tract and acting as an important tool to inhibit pathogenic bacteria. Peptostreptococcaceae is another important group of bacteria that produce butyric acid [47,48]. This acid is associated with better nutrient absorption [49], stimulating the growth of intestinal mucosa cells, improving the retention of calcium and phosphorus in the diet, mitigating the coccidiosis challenge [5] and is an important energy source for enterocytes [50,51]. The use of the blend kept this population stable during the challenge, while this population was reduced in the other treatments. The blend also maintained a stable Akkermansiaceae family. Bacteria of the genus *Akkermansia* can use mucus as a source of carbon and nitrogen, produce acetate and propionate, and produce better intestinal health, with an inverse relationship between their relative abundance and intestinal disorders [52–54].

Gharib-Naseri et al. [55] reported that birds challenged by necrotic enteritis showed a reduction in the Ruminococcaceae group, and a similar result was found in this study. The families Lachnospiraceae, Ruminococcaceae, and Erysipelotrichaceae are positively correlated with better feed conversion because they are associated with the production of short-chain fatty acids (SCFA) and degradation of plant materials [56,57]. In this study, the use of the blend increased the abundance of the Erysipelotrichaceae family, with less variation in the relative abundance between non-challenged and challenged birds. This family is also associated with better feed conversion in broilers [58].

In this study it was possible to observe that the non-challenged birds had a greater relative abundance of the Clostridiaceae family, mainly for the monensin and blend groups. Within the Clostridiaceae family, there are different species, including *Clostridium butyricum* and *Clostridium perfringens*. *C*. *butyricum* can produce short-chain fatty acids (e.g., butyric acid) and has been studied as a probiotic in broilers, improving intestinal barrier function and inhibiting pathogens [59]. Huang et al. [60] observed that *C*. *butyricum* decreased the abundance of *C*. *perfringens* in the gut during the development of necrotic enteritis in broilers. *C*. *perfringens* is the most important pathogenic strain capable of producing more than 16 toxins with different modes of action [61] toxin is responsible for hemolysis, tissue necrosis, epithelial barrier dysfunction, and severe inflammation. Consequently it causes rapid loss of performance and high mortality [62–64]. *Eimeria* infection causes damage to enterocytes releasing cellular proteins, and stimulating mucogenesis, which creates a favorable environment for the reproduction of *C*. *perfringens* [62,64,65]. In previously published data using the qRT-PCR technique, it was demonstrated that supplementation with Blend reduced (P <0.05) the number of copies of *C*. *perfringens* compared to the monensin and control groups [26].

The use of antimicrobials, including monensin, can delay the maturation of the intestinal microbiota, consequently affecting the development of the bird’s intestinal immune system and negatively affecting the bird’s health [66,67]. Monensin, when used alone, causes *Lactobacillus* and *Enterococcus* depletion, and when monensin is associated with other antimicrobials, it increases *Escherichia coli* populations [8]. Some studies report that antimicrobials can reduce microbial diversity in the intestine, with a tendency to increase the production of butyrate and lactic acid producing bacteria. In contrast, they reduce bile salt-degrading bacteria, which are responsible for the utilization of carbohydrates and lipids in the diet, and better energy balance [66–68].

Based on previous results [25,26] and this study the use of the blend kept the intestinal microbiota more stable, mitigating the impacts of *Eimeria* spp. challenge. The blend acted as a modulator, mainly for gram-positive bacteria, contributing to a better weight gain and feed conversion rate (P > 0.05) after 14 days of the challenge [26]. The relationship between the immune system and the microbiota is complex, so two hypotheses were created for the mechanism of action of the blend in intestinal health. The first hypothesis is that it acts as a modulator of the intestinal microbiota and these bacteria modulate the gene expression of interleukins, toll-like receptors, and T cells during the parasite's infectious process. The second hypothesis is that blend acts on the immune system during the peak of the inflammatory process caused by the challenge, directing the response against pathogenic bacteria and modulating the microbiota.

## Conclusions

The blend of functional oils besides stimulating the beneficial microbiota, mitigated the impact of a coccidial challenge in broilers. This resulted in greater microbial stability when compared to treatment with the ionophore, monensin.

## Supporting information

S1 Fig Rarefaction curve of the eigthteen samples of the experiment composed of three feed additives, basal diet (control), sodium monensin (Mone), or Blend (Blend) and sanitary challenge (CH) or unchallenged (UN) with coccidiosis. Horizontal axis: the amount of effective sequencing data (rarefaction depth); vertical axis: the observed number of variations of sequence amplification (ASVs).
(PDF)

S1 Table The number of reads that passed through each step of the quality control for the eighteen samples of the experiment composed of three feed additives, basal diet (control), sodium monensin (Mone), or Blend (Blend) and sanitary challenge (CH) or unchallenged (UN) with coccidiosis. (DOCX)

S2 Table Alpha diversity of the intestinal microbiota of broilers at the family level. The experiment is composed of three feed additives, basal diet (control), sodium Monensin (Mone), or Blend (Blend) and sanitary challenge (CH) or unchallenged (UN) with coccidiosis.
(DOCX)

S3 Table Relative abundance of phylum, class, order, family, and genera present in the gut microbiota of broilers. (DOCX)

S1 File (PDF)

S2 File (PDF)
