# Supplementary figures and images for: Modulation of the intestinal microbiota of broilers supplemented with monensin or functional oils in response to challenge by Eimeria spp
Source: PLoS One. 2020 Aug 7;15(8):e0237118. doi: 10.1371/journal.pone.0237118 (PMC7413546; doi:10.1371/journal.pone.0237118)

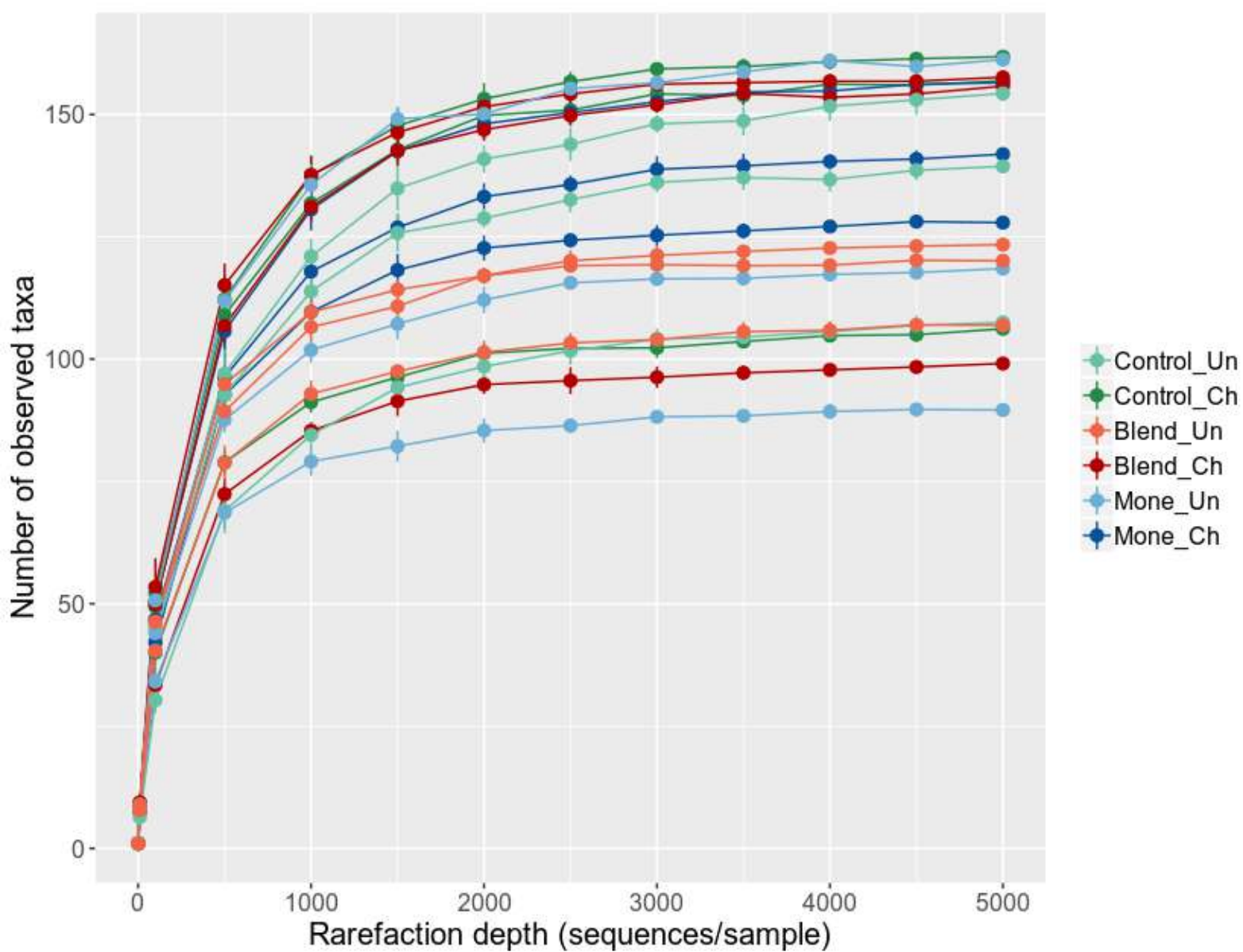

Supplement: S1 Fig — Horizontal axis: the amount of effective sequencing data (rarefaction depth); vertical axis: the observed number of variations of sequence amplification (ASVs). (PDF) [file pone.0237118.s001.pdf]
