# Supplementary material for: Modulation of the intestinal microbiota of broilers supplemented with monensin or functional oils in response to challenge by Eimeria spp
Source: PLoS One. 2020 Aug 7;15(8):e0237118. doi: 10.1371/journal.pone.0237118 (PMC7413546; doi:10.1371/journal.pone.0237118)
Supplement: S1 Table — (DOCX) [file pone.0237118.s002.docx]

**S1 Table.**  The number of reads that passed through each step of the quality control for the eighteen samples of the experiment composed of three feed additives, basal diet (control), sodium Monensin (Mone), or Blend (Blend) and sanitary challenge (CH) or unchallenged (UN) with coccidiosis.

| **Samples** | **Input** | **Filtered** | **Denoised** | **Non-chimeric** | **Non-rare** |
| --- | --- | --- | --- | --- | --- |
| **Control_Ch** | 49908 | 45378 | 45378 | 45168 | 44640 |
| **Control_Ch** | 51289 | 47093 | 47093 | 46318 | 45544 |
| **Control_Ch** | 41824 | 37939 | 37939 | 37939 | 37390 |
| **Mone_Ch** | 55928 | 51536 | 51536 | 49683 | 48936 |
| **Mone_Ch** | 37923 | 34977 | 34977 | 32834 | 31974 |
| **Mone_Ch** | 48632 | 43428 | 43428 | 43409 | 42635 |
| **Blend_Ch** | 47474 | 43545 | 43545 | 39758 | 39167 |
| **Blend_Ch** | 45298 | 41315 | 41315 | 39115 | 38248 |
| **Blend_Ch** | 53148 | 48911 | 48911 | 46218 | 45368 |
| **Control_Un** | 52792 | 48679 | 48679 | 46867 | 46374 |
| **Control_Un** | 47686 | 42676 | 42676 | 42350 | 41595 |
| **Control_Un** | 63005 | 57993 | 57993 | 54091 | 53276 |
| **Mone_Un** | 33988 | 31341 | 31341 | 31096 | 30901 |
| **Mone_Un** | 58916 | 52625 | 52625 | 50611 | 49749 |
| **Mone_Un** | 42404 | 38753 | 38753 | 38691 | 38329 |
| **Blend_Un** | 37861 | 34508 | 34508 | 34181 | 33792 |
| **Blend_Un** | 43041 | 39348 | 39348 | 38370 | 37692 |
| **Blend_Un** | 25672 | 23435 | 23435 | 23409 | 22852 |
| **Total** |  |  |  |  | **728,462** |
| **Average** |  |  |  |  | **40,470.11** |
| **SD** |  |  |  |  | **7,580.40** |
