# Supplementary material for: Modulation of the intestinal microbiota of broilers supplemented with monensin or functional oils in response to challenge by Eimeria spp
Source: PLoS One. 2020 Aug 7;15(8):e0237118. doi: 10.1371/journal.pone.0237118 (PMC7413546; doi:10.1371/journal.pone.0237118)
Supplement: S2 Table — The experiment is composed of three feed additives, basal diet (control), sodium Monensin (Mone), or Blend (Blend) and sanitary challenge (CH) or unchallenged (UN) with coccidiosis. (DOCX) [file pone.0237118.s003.docx]

**S2 Table****.** Alpha diversity of the intestinal microbiota of broilers at the family level. The experiment is composed of three feed additives, basal diet (control), sodium Monensin (Mone), or Blend (Blend) and sanitary challenge (CH) or unchallenged (UN) with coccidiosis.

| **Treatment** | **Chao1** | **Shannon** | **Simpson** |
| --- | --- | --- | --- |
| **Control_Ch** | 20.00 ± 2.65 | 1.50 ± 0.13 | 0.70 ± 0.05 |
| **Control_Un** | 18.00 ± 1.73 | 1.09 ± 0.19 | 0.56 ± 0.10 |
| **Blend_Ch** | 18.33 ± 1.53 | 1.39 ± 0.07 | 0.66 ± 0.02 |
| **Blend_Un** | 19.00 ± 3.46 | 1.42 ± 0.08 | 0.68 ± 0.02 |
| **Mone_Ch** | 20.67 ± 3.06 | 1.50 ± 0.17 | 0.69 ± 0.05 |
| **Mone_Un** | 19.00 ± 2.65 | 1.19 ± 0.15 | 0.61 ± 0.05 |
| ***p* value** | 0.714 | 0.040 | 0.399 |
| **chi-squared** | 2.909 | 11.667 | 5.140 |

Chao = number of families present in the samples. Shannon = abundance and evenness of the families present in the samples. Simpson = number and abundance of each family present. *p* values lower than 0.05 imply significant differences by non-parametric Kruskal-walis test. Values are means and standard deviation of 3 replicates and a pool of 8 birds/replicate.
