# Supplementary material for: Modulation of the intestinal microbiota of broilers supplemented with monensin or functional oils in response to challenge by Eimeria spp
Source: PLoS One. 2020 Aug 7;15(8):e0237118. doi: 10.1371/journal.pone.0237118 (PMC7413546; doi:10.1371/journal.pone.0237118)
Supplement: S3 Table — (DOCX) [file pone.0237118.s004.docx]

**S3 Table.** **Relative abundance of phylum, class, order, family, and genera present in the gut microbiota of broilers.**

| **Phylum** | **Control_Ch** | **Mone_Ch** | **Blend_Ch** | **Control_Un** | **Mone_Un** | **Blend_Un** | **chi-squared** | ***p* value** |
| --- | --- | --- | --- | --- | --- | --- | --- | --- |
| **Actinobacteria** | 0,26±0,20 | 0,34±0,10 | 0,18±0,02 | 0,15±0,03 | 0,39±0,35 | 0,33±0,28 | 4,42 | 0,49 |
| **Bacteroidetes** | 0,80±0,22 | 2,02±1,48 | 1,92±1,59 | 0,88±0,64 | 0,87±0,56 | 0,71±0,99 | 4,13 | 0,53 |
| **Cyanobacteria** | 0,04±0,07 | 0,03±0,05 | 0,02±0,03 | 0,07±0,13 | - | - | 2,49 | 0,78 |
| **Firmicutes** | 96,00±0,64 | 94,07±2,87 | 95,64±2,28 | 96,59±0,32 | 97,05±1,56 | 95,63±0,51 | 4,84 | 0,44 |
| **Proteobacteria** | 0,28±0,24 | 0,84±0,43 | 0,23±0,18 | 0,65±0,85 | 0,52±0,17 | 0,64±0,26 | 7,08 | 0,21 |
| **Tenericutes** | 2,51±0,63 | 1,30±0,20 | 1,91±1,48 | 1,60±1,21 | 1,10±1,17 | 2,60±1,72 | 4,16 | 0,53 |
| **Verrucomicrobia** | 0,10±0,13 | 1,41±2,37 | 0,10±0,16 | 0,01±0,01 | 0,07±0,10 | 0,10±0,17 | 2,75 | 0,74 |
| **Unknown** | - | - | - | 0,05±0,08 | - | - | 5,00 | 0,42 |
| **Class** |  |  |  |  |  |  |  |  |
| **Actinobacteria** | - | - | - | - | - | 0,10±0,17 | 5,00 | 0,42 |
| **Coriobacteriia** | 0,26±0,20 | 0,34±0,10 | 0,18±0,02 | 0,15±0,03 | 0,39±0,35 | 0,23±0,12 | 5,12 | 0,40 |
| **Bacteroidia** | 0,80±0,22 | 2,02±1,48 | 1,92±1,59 | 0,88±0,64 | 0,87±0,56 | 0,71±0,99 | 4,13 | 0,53 |
| **Melainabacteria** | 0,04±0,07 | 0,03±0,05 | 0,02±0,03 | 0,07±0,13 | - | - | 2,49 | 0,78 |
| **Bacilli** | 15,84±5,73 | 12,72±6,54 | 12,49±7,39 | 9,40±13,92 | 6,94±6,57 | 16,70±3,03 | 4,30 | 0,51 |
| **Clostridia** | 75,08±6,38 | 78,74±9,53 | 78,95±6,32 | 84,50±15,28 | 88,43±7,51 | 76,40±3,17 | 4,86 | 0,43 |
| **Erysipelotrichia** | 5,01±0,59 | 2,46±0,20 | 4,13±1,30 | 2,65±1,67 | 1,61±0,91 | 2,45±0,40 | 10,73 | 0,06 |
| **Negativicutes** | 0,07±0,06 | 0,15±0,04 | 0,06±0,03 | 0,03±0,05 | 0,06±0,06 | 0,07±0,04 | 8,19 | 0,15 |
| **Alphaproteobacteria** | 0,06±0,10 | - | - | - | - | - | 5,00 | 0,42 |
| **Deltaproteobacteria** | - | - | - | - | 0,04±0,04 | 0,02±0,04 | 7,86 | 0,16 |
| **Gammaproteobacteria** | 0,23±0,13 | 0,84±0,43 | 0,23±0,18 | 0,65±0,85 | 0,47±0,19 | 0,62±0,22 | 9,28 | 0,10 |
| **Mollicutes** | 2,51±0,63 | 1,30±0,20 | 1,91±1,48 | 1,60±1,21 | 1,10±1,17 | 2,60±1,72 | 4,16 | 0,53 |
| **Verrucomicrobiae** | 0,10±0,13 | 1,41±2,37 | 0,10±0,16 | 0,01±0,01 | 0,07±0,10 | 0,10±0,17 | 2,75 | 0,74 |
| **Unknown** | - | - | - | 0,05±0,08 | - | - | 5,00 | 0,42 |
| **Order** |  |  |  |  |  |  |  |  |
| **Bifidobacteriales** | - | - | - | - | - | 0,10±0,17 | 5,00 | 0,42 |
| **Coriobacteriales** | 0,26±0,20 | 0,34±0,10 | 0,18±0,02 | 0,15±0,03 | 0,39±0,35 | 0,23±0,12 | 5,12 | 0,40 |
| **Bacteroidales** | 0,80±0,22 | 2,02±1,48 | 1,92±1,59 | 0,88±0,64 | 0,87±0,56 | 0,71±0,99 | 4,13 | 0,53 |
| **Gastranaerophilales** | 0,04±0,07 | 0,03±0,05 | 0,02±0,03 | 0,07±0,13 | - | - | 2,49 | 0,78 |
| **Bacillales** | 0,05±0,09 | 0,04±0,07 | 0,02±0,03 | 0,04±0,06 | - | - | 2,49 | 0,78 |
| **Lactobacillales** | 15,79±5,82 | 12,68±6,47 | 12,48±7,43 | 9,36±13,85 | 6,94±6,57 | 16,70±3,03 | 4,30 | 0,51 |
| **Clostridiales** | 75,08±6,38 | 78,74±9,53 | 78,95±6,32 | 84,50±15,28 | 88,43±7,51 | 76,40±3,17 | 4,86 | 0,43 |
| **Erysipelotrichales** | 5,01±0,59 | 2,46±0,20 | 4,13±1,30 | 2,65±1,67 | 1,61±0,91 | 2,45±0,40 | 10,73 | 0,06 |
| **Selenomonadales** | 0,07±0,06 | 0,15±0,04 | 0,06±0,03 | 0,03±0,05 | 0,06±0,06 | 0,07±0,04 | 8,19 | 0,15 |
| **Rhodospirillales** | 0,06±0,10 | - | - | - | - | - | 5,00 | 0,42 |
| **Desulfovibrionales** | - | - | - | - | 0,04±0,04 | 0,02±0,04 | 7,86 | 0,16 |
| **Betaproteobacteriales** | - | 0,04±0,07 | - | 0,01±0,02 | 0,08±0,09 | - | 6,43 | 0,27 |
| **Enterobacteriales** | 0,13±0,03 | 0,69±0,24 | 0,21±0,16 | 0,61±0,86 | 0,34±0,20 | 0,57±0,15 | 8,35 | 0,14 |
| **Pseudomonadales** | 0,09±0,12 | 0,11±0,13 | 0,03±0,03 | 0,03±0,03 | 0,05±0,05 | 0,05±0,08 | 1,77 | 0,88 |
| **Mollicutes RF39** | 2,51±0,63 | 1,30±0,20 | 1,91±1,48 | 1,60±1,21 | 1,10±1,17 | 2,60±1,72 | 4,16 | 0,53 |
| **Verrucomicrobiales** | 0,10±0,13 | 1,41±2,37 | 0,10±0,16 | 0,01±0,01 | 0,07±0,10 | 0,10±0,17 | 2,75 | 0,74 |
| **Unknown** | - | - | - | 0,05±0,08 | - | - | 5,00 | 0,42 |
| **Family** |  |  |  |  |  |  |  |  |
| **Bifidobacteriaceae** | - | - | - | - | - | 0,10±0,17 | 5,00 | 0,42 |
| **Eggerthellaceae** | 0,26±0,20 | 0,34±0,10 | 0,18±0,02 | 0,15±0,03 | 0,39±0,35 | 0,23±0,12 | 5,12 | 0,40 |
| **Bacteroidaceae** | 0,67±0,22 | 1,74±1,12 | 1,53±1,50 | 0,67±0,66 | 0,57±0,53 | 0,67±0,98 | 3,88 | 0,57 |
| **Rikenellaceae** | - | - | - | - | 0,25±0,23 | 0,03±0,04 | 9,70 | 0,08 |
| **Tannerellaceae** | 0,13±0,08 | 0,28±0,36 | 0,39±0,33 | 0,22±0,24 | 0,04±0,08 | 0,01±0,02 | 6,64 | 0,25 |
| **Bacillaceae** | 0,05±0,09 | 0,04±0,07 | 0,02±0,03 | 0,04±0,06 | - | - | 2,49 | 0,78 |
| **Aerococcaceae** | - | - | 0,03±0,05 | - | - | 0,02±0,03 | 4,25 | 0,51 |
| **Enterococcaceae** | 1,13±0,96 | 0,51±0,58 | 0,78±0,99 | 0,10±0,04 | 0,05±0,09 | 0,95±0,93 | 10,23 | 0,07 |
| **Lactobacillaceae** | 13,83±6,39 | 11,01±7,44 | 11,64±6,66 | 9,12±13,97 | 6,53±6,76 | 15,06±2,20 | 3,43 | 0,63 |
| **Streptococcaceae** | 0,83±0,89 | 1,16±1,89 | 0,02±0,03 | 0,14±0,08 | 0,36±0,34 | 0,67±0,16 | 9,34 | 0,10 |
| **Christensenellaceae** | 1,16±1,05 | 1,82±1,25 | 1,21±1,06 | 0,77±0,80 | 0,84±0,63 | 0,75±0,71 | 3,01 | 0,70 |
| **Clostridiaceae 1** | 0,06±0,05 | 0,04±0,04 | 0,02±0,02 | 0,15±0,14 | 0,21±0,13 | 0,23±0,40 | 6,18 | 0,29 |
| **Clostridiales vadinBB60 group** | - | 0,03±0,05 | 0,01±0,02 | - | - | - | 4,25 | 0,51 |
| **Defluviitaleaceae** | 0,18±0,21 | 0,19±0,05 | 0,22±0,15 | 0,02±0,02 | 0,25±0,22 | 0,05±0,06 | 8,71 | 0,12 |
| **Eubacteriaceae** | - | 0,10±0,18 | - | - | - | 0,00±0,01 | 4,25 | 0,51 |
| **Family XIII** | 0,45±0,06 | 0,32±0,11 | 0,39±0,06 | 0,24±0,06 | 0,37±0,06 | 0,44±0,25 | 7,53 | 0,18 |
| **Lachnospiraceae** | 30,32±3,61 | 37,52±6,14 | 37,22±12,33 | 25,26±3,51 | 42,16±1,75 | 30,50±1,83 | 10,33 | 0,07 |
| **Peptococcaceae** | 0,13±0,07 | 0,11±0,04 | 0,09±0,09 | 0,08±0,07 | 0,04±0,04 | 0,07±0,05 | 4,13 | 0,53 |
| **Peptostreptococcaceae** | 1,51±0,86 | 1,10±1,21 | 1,39±0,59 | 0,18±0,19 | 0,47±0,18 | 1,11±0,51 | 10,24 | 0,07 |
| **Ruminococcaceae** | 41,27±8,95 | 37,50±5,59 | 38,38±16,74 | 57,77±11,74 | 44,08±5,63 | 43,25±3,73 | 5,91 | 0,31 |
| **Erysipelotrichaceae** | 5,01±0,59 | 2,46±0,20 | 4,13±1,30 | 2,65±1,67 | 1,61±0,91 | 2,45±0,40 | 10,73 | 0,06 |
| **Acidaminococcaceae** | 0,07±0,06 | 0,15±0,04 | 0,06±0,03 | 0,03±0,05 | 0,06±0,06 | 0,07±0,04 | 8,19 | 0,15 |
| **uncultured** | 0,06±0,10 | - | - | - | - | - | 5,00 | 0,42 |
| **Desulfovibrionaceae** | - | - | - | - | 0,04±0,04 | 0,02±0,04 | 7,86 | 0,16 |
| **Burkholderiaceae** | - | 0,04±0,07 | - | 0,01±0,02 | 0,08±0,09 | - | 6,43 | 0,27 |
| **Enterobacteriaceae** | 0,13±0,03 | 0,69±0,24 | 0,21±0,16 | 0,61±0,86 | 0,34±0,20 | 0,57±0,15 | 8,35 | 0,14 |
| **Moraxellaceae** | 0,06±0,08 | 0,07±0,08 | 0,02±0,03 | 0,03±0,03 | 0,04±0,04 | - | 3,92 | 0,56 |
| **Pseudomonadaceae** | 0,03±0,04 | 0,04±0,05 | 0,01±0,02 | - | 0,01±0,01 | 0,05±0,08 | 3,14 | 0,68 |
| **uncultured bacterium** | 0,64±0,55 | 0,38±0,31 | 0,38±0,47 | 0,24±0,38 | 0,02±0,03 | 0,25±0,35 | 3,51 | 0,62 |
| **Akkermansiaceae** | 0,10±0,13 | 1,41±2,37 | 0,10±0,16 | 0,01±0,01 | 0,07±0,10 | 0,10±0,17 | 2,75 | 0,74 |
| **Unknown** | 1,91±0,55 | 0,95±0,17 | 1,55±1,12 | 1,48±0,93 | 1,08±1,18 | 2,35±1,72 | 3,29 | 0,66 |
| **Genus** |  |  |  |  |  |  |  |  |
| **Bifidobacterium** | - | - | - | - | - | 0,10±0,17 | 5,00 | 0,42 |
| **CHKCI002** | 0,22±0,19 | 0,29±0,06 | 0,18±0,02 | 0,11±0,02 | 0,32±0,30 | 0,22±0,13 | 4,25 | 0,51 |
| **Gordonibacter** | 0,05±0,04 | 0,06±0,05 | - | 0,04±0,03 | 0,08±0,05 | 0,01±0,01 | 7,00 | 0,22 |
| **Bacteroides** | 0,67±0,22 | 1,74±1,12 | 1,53±1,50 | 0,67±0,66 | 0,57±0,53 | 0,67±0,98 | 3,88 | 0,57 |
| **Alistipes** | - | - | - | - | 0,25±0,23 | 0,03±0,04 | 9,70 | 0,08 |
| **Parabacteroides** | 0,13±0,08 | 0,28±0,36 | 0,39±0,33 | 0,22±0,24 | 0,04±0,08 | 0,01±0,02 | 6,64 | 0,25 |
| **Bacillus** | 0,05±0,09 | 0,04±0,07 | 0,02±0,03 | 0,04±0,06 | - | - | 2,49 | 0,78 |
| **Globicatella** | - | - | 0,03±0,05 | - | - | 0,02±0,03 | 4,25 | 0,51 |
| **Enterococcus** | 1,13±0,96 | 0,51±0,58 | 0,78±0,99 | 0,10±0,04 | 0,05±0,09 | 0,95±0,93 | 10,23 | 0,07 |
| **Lactobacillus** | 13,83±6,39 | 11,01±7,44 | 11,64±6,66 | 9,12±13,97 | 6,53±6,76 | 15,06±2,20 | 3,43 | 0,63 |
| **Streptococcus** | 0,83±0,89 | 1,16±1,89 | 0,02±0,03 | 0,14±0,08 | 0,36±0,34 | 0,67±0,16 | 9,34 | 0,10 |
| **Christensenellaceae R-7 group** | 1,16±1,05 | 1,82±1,25 | 1,21±1,06 | 0,77±0,80 | 0,84±0,63 | 0,75±0,71 | 3,01 | 0,70 |
| **Candidatus Arthromitus** | 0,06±0,05 | 0,04±0,04 | 0,02±0,02 | 0,11±0,17 | 0,21±0,13 | 0,23±0,40 | 4,82 | 0,44 |
| **uncultured Clostridia bacterium** | - | 0,03±0,05 | 0,01±0,02 | - | - | - | 4,25 | 0,51 |
| **Defluviitaleaceae UCG-011** | 0,18±0,21 | 0,19±0,05 | 0,22±0,15 | 0,02±0,02 | 0,25±0,22 | 0,05±0,06 | 8,71 | 0,12 |
| **Eubacterium** | - | 0,10±0,18 | - | - | - | 0,00±0,01 | 4,25 | 0,51 |
| **Family XIII AD3011 group** | 0,12±0,04 | 0,09±0,03 | 0,08±0,01 | 0,03±0,00 | - | 0,08±0,09 | 10,92 | 0,05 |
| **Family XIII UCG-001** | 0,02±0,03 | - | 0,03±0,03 | 0,01±0,01 | 0,02±0,04 | - | 4,15 | 0,53 |
| **[Eubacterium] hallii group** | 0,17±0,06 | 0,21±0,08 | 0,07±0,12 | 0,14±0,04 | 0,30±0,14 | 0,08±0,14 | 6,23 | 0,28 |
| **[Ruminococcus] torques group** | 5,38±0,75 | 10,79±2,85 | 12,54±8,07 | 4,16±0,28 | 1,62±1,58 | 4,78±2,18 | 13,58 | 0,02 |
| **Blautia** | 2,44±0,56 | 1,52±0,86 | 1,62±1,30 | 2,98±2,68 | 4,29±3,34 | 1,29±0,72 | 4,74 | 0,45 |
| **CHKCI001** | 0,09±0,15 | 0,17±0,11 | 0,08±0,07 | 0,04±0,03 | 0,16±0,17 | 0,04±0,07 | 4,81 | 0,44 |
| **GCA-900066575** | 0,06±0,10 | 0,05±0,08 | 0,12±0,21 | - | - | 0,08±0,13 | 2,49 | 0,78 |
| **Lachnoclostridium 5** | 0,04±0,06 | - | - | - | - | - | 5,00 | 0,42 |
| **Sellimonas** | - | - | - | 0,08±0,14 | - | - | 5,00 | 0,42 |
| **Tyzzerella** | 0,17±0,16 | 0,17±0,16 | 0,34±0,15 | 0,29±0,35 | 0,49±0,54 | 0,14±0,12 | 3,22 | 0,67 |
| **Tyzzerella 3** | 0,08±0,08 | - | 0,10±0,09 | 0,13±0,11 | 0,32±0,22 | 0,18±0,17 | 6,92 | 0,23 |
| **Lachnospiraceae_uncultured** | - | 0,05±0,08 | 0,12±0,21 | - | - | - | 4,25 | 0,51 |
| **Peptococcaceae_uncultured** | 0,13±0,07 | 0,11±0,04 | 0,09±0,09 | 0,10±0,07 | 0,04±0,04 | 0,07±0,05 | 4,13 | 0,53 |
| **Romboutsia** | 1,51±0,86 | 1,10±1,21 | 1,39±0,59 | 0,03±0,05 | 0,16±0,18 | 0,75±0,42 | 12,33 | 0,03 |
| **[Eubacterium] coprostanoligenes group** | 2,86±1,11 | 3,53±1,62 | 2,22±1,41 | 3,24±1,65 | 2,82±1,00 | 3,14±0,86 | 1,09 | 0,95 |
| **Anaerofilum** | 0,09±0,16 | 0,18±0,31 | - | - | - | - | 4,25 | 0,51 |
| **Anaerotruncus** | 0,07±0,08 | - | 0,11±0,15 | - | 0,03±0,06 | - | 7,37 | 0,19 |
| **Butyricicoccus** | 0,90±0,57 | 0,62±0,25 | 1,30±0,55 | 0,71±0,50 | 1,69±1,01 | 0,58±0,22 | 5,51 | 0,36 |
| **CAG-352** | 0,01±0,01 | 0,17±0,15 | - | - | - | - | 8,41 | 0,14 |
| **DTU089** | 0,30±0,16 | 0,43±0,21 | 0,31±0,18 | 0,32±0,20 | 0,40±0,18 | 0,26±0,13 | 1,89 | 0,86 |
| **Faecalibacterium** | 9,82±4,42 | 7,95±5,35 | 6,70±5,72 | 9,26±4,75 | 4,92±3,55 | 6,75±2,76 | 2,89 | 0,72 |
| **Fournierella** | 0,22±0,28 | 0,06±0,10 | 0,19±0,07 | 0,05±0,05 | - | - | 9,07 | 0,11 |
| **Intestinimonas** | - | - | 0,17±0,29 | - | - | - | 5,00 | 0,42 |
| **Negativibacillus** | 0,30±0,11 | 0,67±0,30 | 0,22±0,09 | 0,27±0,08 | 0,37±0,45 | 0,18±0,06 | 6,66 | 0,25 |
| **Oscillibacter** | - | 0,04±0,07 | 0,09±0,08 | 0,08±0,08 | 0,14±0,13 | - | 6,53 | 0,26 |
| **Ruminiclostridium 5** | 0,93±0,31 | 1,10±0,21 | 1,17±0,75 | 0,94±0,10 | 1,20±0,12 | 0,76±0,05 | 6,17 | 0,29 |
| **Ruminococcaceae NK4A214 group** | 0,53±0,36 | 0,35±0,18 | 1,52±1,33 | 0,46±0,41 | 0,43±0,51 | 0,34±0,36 | 1,56 | 0,91 |
| **Ruminococcaceae UCG-004** | 0,27±0,04 | 0,28±0,08 | 0,27±0,12 | 0,23±0,08 | 0,41±0,19 | 0,27±0,14 | 2,38 | 0,79 |
| **Ruminococcaceae UCG-005** | 0,19±0,17 | 0,48±0,16 | 0,60±0,75 | 0,35±0,11 | 0,28±0,40 | 0,15±0,10 | 5,10 | 0,40 |
| **Ruminococcaceae UCG-009** | 0,01±0,02 | 0,01±0,02 | 0,02±0,03 | 0,01±0,02 | 0,11±0,03 | 0,03±0,04 | 8,70 | 0,12 |
| **Ruminococcaceae UCG-010** | 0,05±0,09 | 0,07±0,06 | 0,08±0,08 | 0,16±0,14 | 0,01±0,01 | 0,03±0,03 | 2,95 | 0,71 |
| **Ruminococcaceae UCG-013** | 1,42±1,05 | 1,54±0,61 | 0,91±0,16 | 1,09±1,21 | 1,19±0,47 | 0,97±0,77 | 1,33 | 0,93 |
| **Ruminococcaceae UCG-014** | 14,82±5,81 | 8,02±0,84 | 11,23±8,34 | 12,73±6,43 | 8,05±3,37 | 12,45±1,21 | 6,05 | 0,30 |
| **Ruminococcus 1** | 0,01±0,02 | 0,04±0,04 | 0,04±0,06 | 0,06±0,10 | 0,03±0,05 | 0,02±0,04 | 0,63 | 0,99 |
| **Ruminococcus 2** | 0,08±0,14 | 0,02±0,03 | - | - | - | - | 4,25 | 0,51 |
| **Subdoligranulum** | 6,32±0,52 | 4,96±0,97 | 5,63±2,86 | 25,47±19,39 | 15,69±10,19 | 14,11±5,09 | 12,70 | 0,03 |
| **Ruminococcaceae_uncultured** | 0,19±0,15 | 0,05±0,09 | 0,20±0,08 | 0,03±0,04 | 0,07±0,12 | - | 9,71 | 0,08 |
| **Erysipelatoclostridium** | 3,43±1,13 | 1,70±0,38 | 2,70±0,21 | 2,37±1,76 | 1,36±0,78 | 1,78±0,21 | 9,16 | 0,10 |
| **Merdibacter** | 0,07±0,01 | 0,19±0,06 | 0,16±0,09 | 0,16±0,13 | 0,20±0,18 | 0,19±0,22 | 1,98 | 0,85 |
| **Turicibacter** | 0,53±0,50 | 0,14±0,16 | 0,37±0,15 | 0,04±0,04 | 0,01±0,01 | 0,40±0,25 | 11,27 | 0,05 |
| **Phascolarctobacterium** | 0,07±0,06 | 0,15±0,04 | 0,06±0,03 | 0,03±0,05 | 0,06±0,06 | 0,07±0,04 | 8,19 | 0,15 |
| **Azospirillum sp. 47_25** | 0,06±0,10 | - | - | - | - | - | 5,00 | 0,42 |
| **Bilophila** | - | - | - | - | 0,04±0,04 | 0,02±0,04 | 7,86 | 0,16 |
| **Parasutterella** | - | 0,04±0,07 | - | 0,01±0,02 | 0,08±0,09 | - | 6,43 | 0,27 |
| **Escherichia-Shigella** | 0,13±0,03 | 0,67±0,20 | 0,21±0,16 | 0,61±0,86 | 0,34±0,20 | 0,54±0,10 | 8,35 | 0,14 |
| **Acinetobacter** | 0,06±0,08 | 0,07±0,08 | 0,02±0,03 | 0,03±0,03 | 0,04±0,04 | - | 3,92 | 0,56 |
| **Pseudomonas** | 0,03±0,04 | 0,04±0,05 | 0,01±0,02 | - | 0,01±0,01 | 0,05±0,08 | 3,14 | 0,68 |
| **uncultured bacterium** | 0,64±0,55 | 0,38±0,31 | 0,38±0,47 | 0,24±0,38 | 0,02±0,03 | 0,25±0,35 | 3,51 | 0,62 |
| **Akkermansia** | 0,10±0,13 | 1,41±2,37 | 0,10±0,16 | 0,01±0,01 | 0,07±0,10 | 0,10±0,17 | 2,75 | 0,74 |
| **Unknown** | 26,99±3,38 | 33,14±5,69 | 30,37±5,69 | 21,70±1,01 | 43,00±3,26 | 30,30±1,47 | 13,35 | 0,02 |

Relative abundance data are expressed as mean ± standard deviation of 3 replicates and a pool of 8 birds/replicate.

p values and chi-squared by non-parametric Kruskal-walis test.
