# Supplementary material for: Modulation of the intestinal microbiota of broilers supplemented with monensin or functional oils in response to challenge by Eimeria spp
Source: PLoS One. 2020 Aug 7;15(8):e0237118. doi: 10.1371/journal.pone.0237118 (PMC7413546; doi:10.1371/journal.pone.0237118)
Supplement: S1 File — (PDF) [file pone.0237118.s005.pdf]

Comparison between a commercial blend of functional oils and monensin on the performance and microbiota of coccidiosis-challenged broilers

P. O. Moraes,*,[1] K. M. Cardinal,† F. L. Gouvêa,† B. Schroeder,† M. S. Ceron,§ R. Lunedo,‡ A. P. G. Frazzon,† J. Frazzon,† and A. M. L. Ribeiro†

**Universidade Federal de Santa Catarina, Florianópolis, RS 88034-000, Brazil; †Universidade Federal do Rio Grande do Sul, Porto Alegre, RS 91540-000, Brazil; ‡Universidade Estadual Paulista Júlio de Mesquita Filho, Pres. Prudente, SP 19014-020, Brazil; and §Universidade José do Rosário Vellano (Unifenas), Alfenas, MG 37132440, Brazil*

Downloaded from https://academic.oup.com/ps/advance-article-abstract/doi/10.3382/ps/pez345/5524378 by Nottingham Trent University user on 01 July 2019

ABSTRACT The aim of the study was to evaluate the effects of a cashew nut shell oil and commercial castor oil blend (CNSL-Castor oil) on the performance and microbiota of broiler chickens with and without coccidiosis challenge. A total of 864 one-day-old male chicks (Cobb) were randomly distributed to receive 6 treatments (8 pens/treatment; 18 chicks/pen) in a 3 × 2 factorial, with 3 additives (control [non-additives], 100 ppm sodium monensin, or 0.15% CNSL-Castor oil blend), and 2 levels of coccidiosis challenge at 14 D of age (unchallenged or inoculated by gavage with 1 mL of solution containing oocysts sporulated with *Eimeria tenella, Eimeria acervulina*, and *Eimeria maxima*). No differences in productive performance were observed among treatments in the pre-challenge period and in unchallenged birds ($P > 0.05$). Seven-days post-challenge, birds receiving monensin performed better than birds in the positive control group (non-additive and challenge) or in the CNSL-Castor oil group ($P > 0.05$). However, 14 D post-challenge, birds supplemented with CNSL-Castor oil presented higher weight gain and better feed conversion ($P > 0.05$), without any change in feed intake ($P > 0.05$). During the accumulated period (1 to 42 D of age), the live weight, weight gain, and feed intake did not differ between the CNSL-Castor oil and monensin groups, both of which presented higher values than the positive control. *Lactobacillus* spp. and *Clostridium perfringens* numbers were increased in the challenged birds ($P < 0.05$). CNSL-Castor oil supplementation reduced *Clostridium* cluster XIV, *C. perfringens,* and *S. aureus*, compared with the monensin and control groups ($P > 0.05$). In addition, the CNSL-Castor oil group presented the highest number of Lactobacillus spp. copies, followed by the monensin and positive control groups ($P > 0.05$). Thus, monensin and CNSL-Castor oil effectively minimized the impact of coccidiosis at different times. While monensin acts as an antimicrobial, CNSL-Castor oil modulates the intestinal microbiota with antimicrobial action against gram-positive bacteria, mainly *C. perfringens* and *S. aureus.*

Key words: Coccidiosis, functional oil, gut health, microbiota, monensin

2019 Poultry Science 0:1–9
http://dx.doi.org/10.3382/ps/pez345

INTRODUCTION

The microbiota evolves with the host as a mutualistic partner, and its balance is linked to the abundance and diversity of species. However, dysbiosis can cause disorders that affect intestinal wall morphology, reduce diversity by increasing the pathogenic bacteria population, induce an immune response, divert energy and nutrients from growth to the inflammatory response, and consequently reduce performance (DiAngelo et al., 2009; Kogut, 2013). Therefore, there is great interest in the development of feed additives that can improve performance, control pathogens, and modulate intestinal microbiota. The phytogenetics have presented interesting results improving intestinal health and modulating the microbiota as additives for animal production (Hume et al., 2006; Oviedo-Rondón et al., 2006; Oviedo-Rondón et al., 2010; Abdel-Wareth et al., 2012; Kley et al., 2012; Kim et al., 2013).

Factors such as age, diet, feed additives, and presence of pathogens alter the intestinal microbiota. Coccidiosis challenge can markedly change the bacterial community in the gut, reducing microbial diversity (Kley et al., 2012) and creating a favorable environment for the dissemination of pathogens, such as the gram-positive bacteria *Clostridium perfringens* (Baba et al., 1997). Oviedo-Rondón et al. (2010) reported that the microbial profile in the ileum and cecum was altered by 45 and 64%, respectively, after *Eimeria* spp. challenge.

© 2019 Poultry Science Association Inc.
Received January 25, 2019.
Accepted June 4, 2019.
[1]Corresponding author: priscila.moraes@ufsc.br

However, using diets supplemented with a phytogenic blend, this change was only 19 and 32%.

Within the phytogenic category, functional oils are defined as oils that have an action beyond nutrition (Murakami et al., 2014). Castor oil is a functional oil composed of 90% ricinoleic acid, and is known for its laxative action (Vieira et al., 2001). In addition, it has antimicrobial action; ester derivatives break the glycosidic bonds of the peptidoglycans present in the walls of gram-positive bacteria (Guimarães et al., 2010).

Cashew nut liquid is mainly composed of cardanol, cardol, and anacardic acid (Mazzetto et al., 2009). The antimicrobial activity of the liquid is associated with the number of terpenoids and phenolic compounds present (Kanehashi et al., 2015), which act against gram-positive bacteria (Parasa et al., 2011). In vitro studies have shown that both functional oils function as ionophores (Vieira et al., 2001; Toyomizu et al., 2003).

Because of its chemical properties, a blend of cashew shell liquid and castor oil has been developed and is known commercially as Essential (Essential, US Patent N°. 8377,485 B2: Oligo Basics Agroind. Ltda., Rua Sérgio Gasparetto 503, Cascavel, PR-CEP, Brazil). The use of this commercial blend in the diet of coccidiosis-challenged broilers resulted in increased weight gain and improved feed conversion (Murakami et al., 2014), as well as an improvement in a 100 kcal of ME (Bess et al., 2012 and Murakami et al., 2014). This increased energy availability may be associated with the antimicrobial effects of the functional oils (Bess et al., 2012); however, no studies have demonstrated its antimicrobial action in vivo.

Anticoccidial drugs are given preventively and continuously in the diet to minimize problems with coccidiosis. The monensin is an ionophore widely used in poultry production; however, *Eimeria* spp. strains resistant to ionophores were already identified (Chapman et al., 2010). In addition, the constant discussion about reducing the use of antibiotics as growth promoters has stimulated the search for alternative methods that can reduce the impact of this parasite and act as growth promoters at the same time.

The aim of the present study was to evaluate the effect of cashew shell liquid and castor oil blend on the performance and microbiota of broilers challenged with coccidiosis, compared with the ionophore monensin.

MATERIALS AND METHODS

All procedures used in this experiment were approved by the Ethics Committee on Animal Use of Federal University of Rio Grande do Sul, under protocol number 29,814, following the legislation for the protection of animals used for scientific purposes (NIH Publications No. 8023, revised 1978).

Animals and Diets

A total of 864 one-day-old male chicks (Cobb 500) were obtained from a commercial hatchery and housed in 2 identical experimental rooms, one for challenged and one for unchallenged birds, thus avoiding cross-contamination. The rooms were composed of 48 pens with an initial density of 18 birds per pen. The nutritional program consisted of 3 diets: pre-starter (1 to 7 D), starter (8 to 21 D), and grower (22 to 28 D), based on the nutritional requirements recommended by the Brazilian Tables of Pigs and Swine (Rostagno et al., 2011). The nutritional composition was the same for all treatments, varying only in the additive used (Table 1).

Table 1. Ingredient formulas and chemical composition of experimental diets according to the rearing period.

Ingredients (%)	Pre Starter (1 to 7 D)	Starter (8 to 21 D)	Grower (22 to 42 D)
Corn	538.55	571.05	597.75
Soybean meal	384.60	354.60	321.10
Vegetal oil	31.80	33.30	43.00
Dicalcium phosphate	19.00	16.60	16.30
Limestone	10.10	10.10	7.80
Salt	5.10	4.80	4.60
L-Lys HCl	3.00	2.50	2.70
DL-Met	3.70	3.20	3.00
L-Ter	1.20	0.80	0.70
Vit-min premix [1]	1.05	1.05	1.05
Choline chloride	0.40	0.50	0.50
Inert/Monensin/CNSL-Castor oil[2]	1.50	1.50	1.50
Total (kg)	1,000.00	1,000.00	1,000.00
Calculated composition			
Metabolizable energy (Kcal/kg)	3,000	3,050	3,150
Crude protein (g/kg)	222.0	210.0	196.9
Calcium (g/kg)	9.2	8.6	7.6
Available P (g/kg)	4.7	4.2	4.1
Digestible P (g/kg)	3.9	3.6	3.5
Potassium (g/kg)	8.6	8.1	7.6
Sodium (g/kg)	2.2	2.1	2.0
Chlorine (g/kg)	3.5	3.4	3.2
Dig. lysine (g/kg)	13.2	2.2	1.5
Dig. methionine (g/kg)	6.5	5.9	5.6
Dig. Met+Cys (g/kg)	9.5	8.8	8.3
Dig. threonine (g/kg)	8.6	7.9	7.3
Dig. tryptophan (g/kg)	2.5	2.3	2.2
Choline (mg/kg)	1,550	1,550	1,450
(Na+K)-Cl (mEq/kg)[3]	216.91	202.59	191.1

[1]Composition (per kg): 150,000 mg of Mn, 100,000 mg of Zn, 80,000 mg of Fe, 15,000 mg of Cu, 1,200 mg of I, 700 mg of Se, 23,200,000 UI of vitamin A, 5,600,000 UI of vitamin D, 52,000 mg of vitamin K, 6000 mg of vitamin B1, 18,000 mg of vitamin B2, 9,000 mg of vitamin B6, 132,000 mg of niacin, 44,000 mg of pantothenic acic, 2,400 mg of folic acid, 200,000 μg of biotin, 40,000 μg of vitamin B12.

[2]At all phases, addition varied according to the treatment (1.50 g/kg of kaolin or CNSL-Castor oil or 0.250 g/kg monensin + 1.25 g/kg of kaolin).

[3]Electrolytic balance.

Each week broilers were weighed, feed intake was measured and in the calculation of feed conversion, the weight of dead birds was considered (Sakomura and Rostagno, 2016).

Experimental Design

The experimental design was completely randomized in a 3 × 2 factorial arrangement: feed additives (basal diet, 100 ppm sodium monensin, or 0.15% CNLS-Castor

Downloaded from https://academic.oup.com/ps/advance-article-abstract/doi/10.3382/ps/pez345/5524378 by Nottingham Trent University user on 01 July 2019

Table 2. Target gene, annealing temperature (TA°C), base pairs, and the ATCC bacterium used for the standard curve, primer sequence, and the reference of the groups and bacterial species studied.

Microorganisms	Target Gene	TA °C	Amp (pb)	ATCC control	Sequence (5′ 3′)	References
Bacteria domain	16S	60	200	*E. coli* (10,536)	F: CGGYCCAGACTCCTACGGG<br>R: TTACCGCGGCTGCTGGCAC	Wise and Siragusa (2007)
Escherichia coli	16S	56	475	*E. coli* (10,536)	F: CCTACGGGAGGCAGCAGT<br>R: CGTTTACGGCGTGGACTAC	Chiang et al. (2006)
Lactobacillus grup	16S	58	341	*L. plantarum* (8014)	F: CACCGCTACACATGGAG<br>R: AGCAGTAGGGAATCTTCCA	Wise and Siragusa (2007)
Staphylococcus aureus	nuc	60	279	*S. aureus* (4163)	F: GCGATTGATGGTGATACGGTT<br>R: AGCCAAGCCTTGACGAACTAAGC	Rinttilä et al. (2004)
Salmonella enteric	invA	58	195	*S. choleraesuis* (10,708)	F: ATTTCAATGGGAACTCTGCC<br>R: ATCGAGATCGCCAATCAGTC	Zhang et al. (2009)
Clostridium cluster XIV	16S	60	116	*C. perfringes* (13,124)	F: ACTCCTACGGGAGGCAGC<br>R: GCTTCTTAGTCARGTACCG	Louie et al. (2012)
Clostridium perfringens	16S	56	120	*C. perfringes* (13,124)	F: ATGCAAGTCGAGCGA(G/T)G<br>R: TATGCGGTATTAATCT(C/T)CCTTT	Rinttilä et al. (2004)
Bifidobacterium spp.	16S	58	437	*B. animalis* (27,672)	F: GGGTGGTAATGCCGGATG<br>R: TAAGCCATGGACTTTCACACC	Bartosh et al. (2005)
Enterococcus spp.	16S	50	124	*E. faecalis* (29,212)	F: GAGAATGATGGAGGTAGAGC<br>R: GACTACGGATCTTATCACTC	Lehner et al. (2005)

oil) and sanitary challenge (challenged or unchallenged with coccidiosis). Both food additives, CNLS-Castor oil and monensin sodium) (Elanco Animal Health, Greenfield, IN), were introduced by replacing inert (kaolin) in the basal diet at all phases.

Challenge and Sample Collection

At 14 D of age, 1 mL of sporulated oocysts of *E. tenella* (10×10^3), *E. acervulina* (200×10^3), and *E. maxima* (80×10^3) was inoculated by gavage. The oocysts were acquired at the *Laboratório de Biologia Molecular de Coccídias* (University of São Paulo/Brazil). Unchallenged chickens received 1 mL of saline, providing the same management stress.

After 7 and 14 D of oocyst inoculation (21 and 28 D of age), 3 birds of average weight from each replicate were euthanized by cervical dislocation and the *Eimeria* spp. lesion score was evaluated. Lesions were ranked from 0 (absence of macroscopic lesions) to 4 (presence of severe macroscopic lesions), according to the method described by Johnson and Reid (1970).

At 28 D of age, intestinal contents were collected from the same birds euthanized to assess lesion score. A portion of 10 cm of the each segment: duodenum segments (from the pylorus exit to the end of the descending duodenal loop), jejunum (descending duodenal loop to Meckel's diverticulum), and ileum (diverticulum to ileocecal insertion) was removed and immediately stored at −20°C.

DNA Extraction

The intestinal contents were separated, and the concentrated bacterial fraction was obtained using the procedure proposed by Apajalahti et al. (1998). DNA was extracted with the PowerFecal DNA Isolation Kit (MoBio, UK), following the manufacturer's recommendations. After extraction, the quality of DNA was verified using a NanoDrop 2000 (Invitrogen) and quantified using Qubit 3.0 (Invitrogen). The DNA obtained was diluted to a concentration of 2 ng/μL.

q-PCR Absolute Curve

The sequence of primers selected, their size, and annealing temperature are shown in Table 2. The reactions were conducted on the StepOnePlus Real-Time PCR System (Applied Biosystems), in a final volume of 15 μL, containing 2.0 μL of PCR buffer 10×; 16 μL of $MgCl_2$ (50 mM) 0.5 μL of each primer (10 μM); 0.2 μL of dNTP (5 mM); 20 μL Sybr green (1×), 0.05 μL Platinum Taq DNA Polymerase (5 U/μL) 5 μL of DNA, and ultrapure water to complete the volume. The conditions for q-PCR were 94°C for 5 min, 35 cycles at 94°C for 30 s, annealing temperature specific for each oligonucleotide pair (Table 2) for 30 s, and 72°C for 30 s. After the amplification cycles, a dissociation curve was obtained for the amplification products by increasing the temperature from 60 to 95°C.

An ATCC bacterium according to the primer (Table 2) was used to construct the standard curve. The bacteria were cultured in specific media without antibiotics. Bacterial genomic DNA was extracted using a PureLink Genomic DNA Kit (Invitrogen). Serial dilutions of DNA were made from 3×10^9 to 3×10^2 on each plate. The threshold was adjusted for each standard curve to achieve an amplification efficiency close to 100%. The cycle threshold (CT) was determined for each sample and compared to the standard curve to determine the number of gene copies in 2 ng of genomic DNA. The number of copies per gram of intestinal

Downloaded from https://academic.oup.com/ps/advance-article-abstract/doi/10.3382/ps/pez345/5524378 by Nottingham Trent University user on 01 July 2019

Table 3. Feed intake (FI), weight gain (WG), and feed conversion ratio (FCR) of unchallenged (UD) and challenged (CD) broilers in the period of 14 to 21 D and 21 to 28 D of age.

	14 to 21 D						21 to 28 D					
	FI (g)		WG (g)		FCR (g/g)		FI (g)		WG (g)		FCR (g/g)	
Treatments	UD	CD	UD	CD	UD	CD	UD	CD	UD	CD	UD	CD
Challenge												
	643 A	486 B	419 A	215 B	1.54 A	2.43 B	870 A	754 B	536 A	396 B	1,63 B	1,95 A
Interaction												
Control	644 Aa	445 Bb	422 Aa	175 Bb	1.52 Aa	2.59 Bb	847 A	741 B	518 Aa	357 Bb	1,63 Ba	2,12 Aa
Monensin	621 Aa	531 Ba	415 Aa	296 Ba	1.52 Aa	1.82 Ba	887 A	741 B	551 Aa	374 Bb	1,61 Ba	2,01 Aa
CNSL-Castor oil [1]	653 Aa	481 Bb	422 Aa	174 Bb	1.55 Aa	2.86 Bb	878 A	780 B	540 Aa	457 Ba	1,64 Ba	1,71 Ab
Mean of additive												
Control	545		298.22 b		2.06 a		794		437 b		1.88 a	
Monensin	581		355.25 a		1.67 b		814		462 b		1.81 a	
CNSL-Castor oil	567		297.62 b		2.21 a		829		499 a		1.68 b	
Probability[2]												
Challenge	***		***		***		***		***		***	
Additive * Challenge	**		***		***		ns		**		***	
Additive	ns		***		***		ns		**		***	
SEM[3]	12.61		13.32		0.1		15.29		19.92		0.04	

Data are expressed as means of the information collected in 144 broilers per treatment. Statistical models included the effects of challenged treatments and interaction.

[1]Essential (US Patent N°. 8377,485; Oligo Basics Ind. Ltda., Cascavel, Paraná, Brazil).

[2]Probabilities: $^{***}P < 0.001$, $^{**}P < 0.05$ and ns: not significant;

[3]SEM: standard error of the mean.

Means with different letters differ statically by LSMEANS, lower case in the column and uppercase in the row within the same variable.

contents was calculated considering the initial mass of the starting material, extraction yield, and the DNA dilution.

Statistical Analysis

The number of gene copies was log10 transformed to obtain a normal distribution. An ANOVA of the factorial arrangement was performed, including the challenge effects, additives, and their interactions for all variables of performance, lesion score, and microbiota. Means were compared by LSmeans when significant differences were found. The GLM procedure of the statistical package SAS, version 9.0 (SAS Institute, 2002) was used.

RESULTS

Growth Performance

In the period before the challenge and for the unchallenged birds, there was no statistical difference in performance among treatments for all periods evaluated. Mortality was less than 1% after challenge (data not shown).

Animal performance was negatively affected by coccidiosis in the first 7-D post-challenge (14 to 21 D of age), decreasing BWG (by 49% and worsening feed conversion ratio (FCR) by 58% ($P < 0.0001$). In the second week post-challenge (21 to 28 D of age), the effect of the negative challenge was less marked, with 26% lower BWG and 20% worse FCR (Table 3). The lowest performance of the challenged birds was also observed during the total rearing period (1 to 42 D of age, Table 4), with lower weight gain, feed intake, and worse feed conversion. The challenged birds exhibited a 17% live weight reduction at 42 D of age.

In the week following the beginning of the challenge (14 to 21 D of age), there was an interaction between additives and challenge for all variables analyzed (Table 3). In the challenged birds, weight gain and feed intake were higher, and feed conversion was better in the monensin group, and no differences were seen between the other groups ($P < 0.05$). Two weeks after the challenge (21 to 28 D of age), birds supplemented with CNLS-Castor oil presented greater weight gain ($P < 0.05$) and better feed conversion ($P < 0.05$) compared with the other treatments. In the whole period (1 to 42 D of age), the live weight of birds in the positive control group was lower than that of birds in the other groups ($P < 0.01$), which showed no differences between them, demonstrating that monensin and CNSL-Castor oil compensated for the negative effect of coccidiosis. No interaction for feed conversion was observed.

When analyzing the factors individually, coccidiosis challenge increased feed conversion, and monensin resulted in better feed conversion when compared to the control birds, regardless of coccidiosis challenge; the effect of CNSL-Castor oil was intermediate, but did not differ from that of the control or monensin.

Lesion Score

In the first week post-infection, broilers receiving monensin had a lower *E. acervulina* lesion score

Downloaded from https://academic.oup.com/ps/advance-article-abstract/doi/10.3382/ps/pez345/5524378 by Nottingham Trent University user on 01 July 2019

Table 4. Body weight (BW), feed intake (FI), weight gain (WG), and feed conversion ratio (FCR) of unchallenged (UD) and challenged (CD) broilers in the period of 1 to 42 D of age.

Treatments	BW (g) UD	BW (g) CD	FI (g) UD	FI (g) CD	WG (g) UD	WG (g) CD	FCR (g/g) UD	FCR (g/g) CD
Challenge								
	2,860 A	2,373 B	4,539 A	3,964 B	2,816 A	2,330 B	1.61 B	1.73 A
Interaction								
Control	2,868 Aa	2,267 Bb	4,597 Aa	3,848 Bb	2,824 Aa	2,225 Ba	1.63	1.73
Monensin	2,873 Aa	2,416 Ba	4,497 Aa	4,038 Ba	2,829 Aa	2,393 Bb	1.59	1.69
CNSL-Castor oil[1]	2,879 Aa	2,435 Ba	4,576 Aa	4,088 Ba	2,836 Aa	2,372 Bb	1.62	1.72
Mean of additive								
Control	2,568 b		4,223		2,525		1.69 a	
Monensin	2,654 a		4,267		2,611		1.62 b	
CNSL-Castor oil [1]	2,628 a		4,332		2,584		1.67 ab	
Probability								
Challenge	***		***		***		**	
Additive * Challenge	**		**		**		Ns	
Additive	**		ns		ns		**	
SEM[3]	44.40		42.28		445.578		0.036	

Data are expressed as means of the information collected in 144 broilers per treatment. Statistical models included the effects of challenged treatments and interaction.

[1]Essential (US Patent N°. 8377,485; Oligo Basics Ind. Ltda., Cascavel, Paraná, Brazil).

[2]Probabilities: $^{***}P < 0.001$, $^{**}P < 0.05$ and ns: not significant;

[3]SEM: standard error of the mean.

Means with different letters differ statically by LSMEANS, lowercase in the column and uppercase in the row within the same variable

compared with the other groups ($P < 0.0001$). In the following week, the broilers with CNSL-Castor oil had a lower *E. tenella* lesion score $P < 0.0480$). There was no difference ($P > 0.05$) among groups for *E. maxima* lesion score in any week evaluated (Figure 1).

Microbiota Modulation Using Monensin or CNSL-Castor oil

There was an interaction among the factors for bacterial domain (total bacteria number), *Lactobacillus* spp., *Clostridium* cluster XIV, *C. perfringens, E. coli,* and *S. aureus* ($P < 0.05$) (Tables 5 and 6). In the challenged birds, monensin reduced the bacterial domain and *E. coli* compared with the other groups. CNSL-Castor oil reduced the copy number of *Clostridium* cluster XIV, *C. perfringens,* and *S. aureus*, with no difference found between monensin and the positive control. In the unchallenged birds, there was no difference in the bacterial domain, *Clostridium* cluster XIV, and *S. aureus* among groups. *Lactobacillus* spp. copy number was lower for the positive control, followed by the monensin and CNSL-Castor oil groups. Both monensin and CNSL-Castor oil reduced the copy number of *C. perfringens* and *E. coli.*

Regardless of the challenge, birds in the positive control group presented more *Bifidobacterium* spp. ($P < 0.05$) copies than birds in the CNSL-Castor oil group, with the monensin group presenting intermediate values. The copy number of *Enterococcus* spp. genus was higher for the positive control group and lower for monensin, and CNSL-Castor oil did not differ.

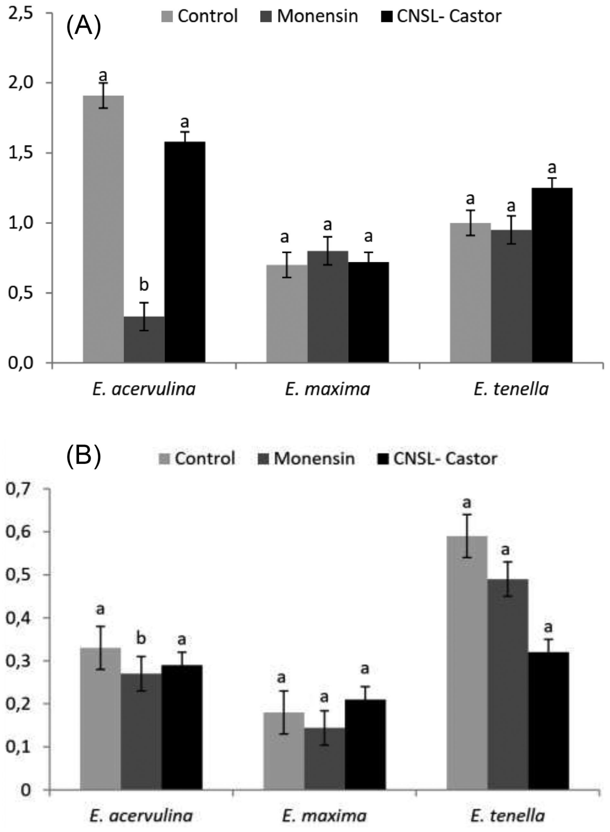

Figure 1. Lesion score in coccidiosis challenged broilers at 21 (A) and 28 (B) days of age—7 and 14 D after challenge. [1] CNSL-Castor (US Patent N. 8377,485; Oligo Basics Ind. Ltda., Cascavel, Paraná, Brazil). Means with different letters differ statically by *LSMeans.*

DISCUSSION

The present study was conducted based on the results of studies published using coccidiosis challenge

Downloaded from https://academic.oup.com/ps/advance-article-abstract/doi/10.3382/ps/pez345/5524378 by Nottingham Trent University user on 01 July 2019

Table 5. Body weight (BW), feed intake (FI), weight gain (WG), and feed conversion ratio (FCR) of unchallenged (UD) and challenged (CD) broilers in the period of 1 to 42 D of age.

Treatments	BW (g)		FI (g)		WG (g)		FCR	
	UD	CD	UD	ND	UD	ND	UD	ND
			Challenge					
	2,860 A	2,373 B	4,539 A	3,964 B	2,816 A	2,330 B	1,61 B	1.73 A
			Interaction					
Control	2,868 Aa	2,267 Bb	4,597 Aa	3,848 Bb	2,824 Aa	2,225 Ba	1.63	1.73
Monensin	2,873 Aa	2,416 Ba	4,497 Aa	4,038 Ba	2,829 Aa	2,393 Bb	1.59	1.69
CNSL-Castor oil[1]	2,879 Aa	2,435 Ba	4,576 Aa	4,088 Ba	2,836 Aa	2,372 Bb	1.62	1.72
			Additive					
Control	2,568 b		4,223		2,525		1.69 a	
Monensin	2,654 a		4,267		2,611		1.62 b	
CNSL-Castor oil [1]	2,628 a		4,332		2,584		1.67 ab	
			Probability[2]					
Challenge	***		***		***		**	
Additive * Challenge	**		**		**		ns	
Additive	**		ns		ns		**	
SEM[3]	44.40		42.28		445.578		0.036	

Data are expressed as means ± standard deviations. Information was collected in 144 broilers per treatment. Statistical models included the effects of challenged treatments and interaction.
[1]Essential (US Patent N°. 8377,485; Oligo Basics Ind. Ltda., Cascavel, Paraná, Brazil).
[2]Probabilities: $^{***}P < 0.001$, $^{**}P < 0.05$ and ns: not significant;
[3]SEM: standard error of the mean.
Means with different letters differ statically by LSMEANS, lowercase in the column and uppercase in the row within the same variable

Table 6. Copy number of *Salmonella enterica, Clostridium perfringens*, *Escherichia coli, Staphylococcus aureus* in the intestinal content of broiler 14 D after challenge with coccidiose.

Treatment	S. enterica		C. perfringens		E.coli		S.aureus	
	UD	CD	UD	CD	UD	CD	UD	CD
			Interaction					
Control	4.17	4.18	6.60 Ba	7.14 Aa	6.63 Aa	6.53 Aa	6.97 Ba	7.39 Aa
Monensin	4.12	4.24	5.28 Bb	6.80 Aa	5.89 Ab	6.00 Ab	6.83 Ba	7.42 Aa
CNSL-Castor oil[1]	4.23	4.31	5.18 Ab	5.27 Ab	5.60 Bb	6.66 Aa	6.77 Aa	6.62 Ab
			Challenge					
Challenge	4.18	4.23	5.50 B	6.81 A	6.00 A	6.10 A	7.05	6.95
			Additives					
Control	4.27		6.62 a		6.07		7.18 a	
Monensin	4.18		5.23 b		5.95		7.13 a	
CNSL-Castor oil[1]	4.25		5.87 b		6.15		6.70 b	
			Probability[2]					
Additive*challenge	0.924		0.0129		0.0308		0.0018	
Challenge	0.6566		0.0115		0.7449		0.3359	
Additive	0.7855		<0.0001		0.8647		0.0009	
SEM[3]	0.145		0.154		0.252		0.0952	

Data are expressed log10 copy number of 16S RNA gene in 1 g of intestinal contents. UD: unchallenged broilers, CD: broilers challenged with coccidiosis. Information represent a pool of intestinal contents of 3 birds per box, totalling an average of 24 birds per treatment. Statistical models included the effects of challenged treatments and interaction.
[1]Essential (US Patent N°. 8377,485; Oligo Basics Ind. Ltda., Cascavel, Paraná, Brazil).
[2]Probabilities: $^{***}P < 0.001$, $^{**}P < 0.05$ and ns: not significant;
[3]SEM: standard error of the mean.
Means with different letters differ statically by LSMEANS, lowercase in the column and uppercase in the row within the same variable

(Cox et al., 2010; Kim et al., 2011; Orengo et al., 2012; Scheurer et al., 2013; Bortoluzzi et al., 2015). In the present study, coccidiosis challenge resulted in irrelevant mortality (< 1%). However, this was sufficient to decrease animal performance throughout the evaluation period.

Anti-coccidial drugs are highly efficient at reducing losses caused by coccidiosis infection. Conversely, intensive use may stimulate parasite resistance (Chapman et al., 2010). Some phytogenetic studies have reported a recovery in performance similar to that observed with the use of drugs (Bess et al., 2012; Kley et al., 2012; Mohiti-Asli and Ghanaatparast-Rashti, 2015). In this study, monensin improved performance in the week immediately post-challenge. However, CNSL-Castor oil provided late but consistent compensatory gains; notably, at 42 D of age, there was no difference in weight gain, feed intake, and live weight between groups.

The worst performance during coccidiosis challenge is associated with reduced intestinal absorption area, nutrient absorption deficit, and inflammation caused in the first week post-challenge (Laurent et al., 2001;

Downloaded from https://academic.oup.com/ps/advance-article-abstract/doi/10.3382/ps/pez345/5524378 by Nottingham Trent University user on 01 July 2019

Cornelissen et al., 2009; Cox et al., 2010). It is possible to speculate that CNSL-Castor oil has lower anticoccidial action than monensin, but its mechanism of action may be associated with the recovery of intestinal health after the inflammation peak, resulting in similar growth performance to that observed with the ionophore during the accumulated period (1 to 42 D).

Intestinal health is directly related to the microbiota profile that interacts with the host. The microbiota regulates the absorptive efficiency, antagonizes the effects of pathogenic bacteria, enhances intestinal integrity, and modulates immunity (Oviedo-Rondón et al., 2010; Pan and Yu, 2014). The results show that coccidiosis challenge did not significantly affect the total number of bacteria, but altered the microbiota profile, increasing the population of *Lactobacillus* spp., *Clostridium* cluster XIV, *C. perfringens,* and *S. aureus*. Some studies have shown an increased population of *Clostridium* bacteria and lactic acid fermenters, such as *Lactobacillus,* following coccidiosis challenge (Kley et al., 2012; M'sadeq et al., 2015; Stanley et al., 2014). This is due to the increased amount of mucus and the presence of proteins from coccidia-damaged cells (Collier et al., 2008), serving as a substrate for beneficial and pathogenic bacteria (Deplancke and Gaskins, 2001).

Unchallenged broilers that received monensin or CNSL-Castor oil exhibited increased levels of *Lactobacillus* and *C. perfringens,* and decreased levels of *E. coli* compared with the control group. *Lactobacillus* have beneficial effects on the host, such as modulating the immune system and antagonizing pathogenic bacteria (Servin 2004; Klose et al., 2010; Yousaf et al., 2017). They are usually considered as a beneficial group; however, the presence of 3 *Lactobacillus* species*, L. salivarius, L. aviaries,* and *L. crispatus*, may be associated with poor performance in broilers, because they deplete bile salts and impair fat emulsification (Guban et al., 2006).

Monensin is an ionophore known for its coccidiostatic, antimicrobial, and growth-promoting action (Huyben et al., 2001). Compared with unchallenged birds receiving monensin, unchallenged birds had lower total bacteria and higher levels of *Clostridium* cluster XVI, *C. perfringens,* and *S. aureus*. This change was similar to the results observed in the positive control group, showing that monensin, even with a reduction in the total number of bacteria, provided a profile similar to the challenged control group. A quantitative and qualitative change in the microbiota profile is characteristic of dysbiosis, which in this case, is caused by coccidiosis. Dysbiosis is defined as an undesirable change in the microbiota, resulting in an imbalance between beneficial and pathogenic bacteria, and may negatively affect animal performance (Ducatelle et al., 2015).

Studies suggest that the use of phytogenic additives positively modulates intestinal microbiota, even in the presence of a coccidiosis challenge (Hume et al., 2006; Oviedo-Rondón et al., 2006; Oviedo-Rondón et al., 2010; Abdel-Wareth et al., 2012; Kley et al., 2012; Kim et al., 2013). In this study, CNSL-Castor oil appeared to be a beneficial modulator of the intestinal microbiota, because it did not cause any differences in the populations of *Lactobacillus* spp., *Clostridium* ssp., *Clostridium perfringens,* and *S. aureus* in challenged broilers, although it did not reduce the total bacteria domain. This balance in the microbiota may have aided the performance recovery after challenge. The microbiota composition may be directly associated with the best animal performance, but how this relationship works is not clear (Stanley et al., 2014). In this study, the population of the *Enterococcus* genus was reduced in challenged animals receiving the functional oil blend. Some species of this genus are pathogenic; for example, *Enterococcus cecorum* is related to bone diseases, such as osteomyelitis (Kense and Landman, 2011). In addition, *Enterococcus* spp. are opportunistic and can spread rapidly when dysbiosis occurs (Cao et al., 2013). Lunedo et al. (2014) associated a worse feed conversion in chickens receiving low tannin sorghum with the increase of *Enterococcus* genus and *Enterobacteriaceae* family in the ileum.

In this study, an increase in the copy number of *S. aureus* species was observed in the challenged broilers, except for the group that received the functional oil blend. In general, the genus *Staphylococcus* spp. is a normal habitant of the skin and mucous membranes and is also considered opportunistic (Jonsson and Wadstrom, 1993). In poultry farming, *S. aureus* infection has been associated with several outbreaks, as these can occur due to management problems or to various infections. Its rapid spread in the intestine occurs when immune resistance is low due to infection by other pathogens, immunosuppression, and skin or mucosal lesions, causing diseases such as salpingitis, folliculitis, bursitis, gangrenous dermatitis, and cellulitis (Ferreira and Ferreira, 2009).

The results of this study show that the functional oil blend has activity against gram-positive bacteria and acts as a modulator of the intestinal microbiota. According to Abbas et al. (2012), the liquid cashew nutshell components, cardol and anacardic acid, have a similar action to a monovalent ionophore, causing damage to the bacterial cell membrane. Moreover, ricinoleic acid has an antimicrobial effect, and denatures and coagulates proteins of the bacterial cell wall. The ester group that composes the ricinoleic acid molecule favors hydrolysis by the plasma esterase that forms alcohol and inhibits the transpeptidase enzyme responsible for the synthesis of peptide glycols (Guimarães et al., 2010). Thus, castor oil may inhibit cell membrane synthesis.

The functional oil blend improved the performance of coccidiosis-challenged broilers in the second week, resulting in similar performance to those receiving

Downloaded from https://academic.oup.com/ps/advance-article-abstract/doi/10.3382/ps/pez345/5524378 by Nottingham Trent University user on 01 July 2019

the ionophore monensin. The blend showed to be a good option under a coccidiosis challenge, acting as a modulator of the intestinal microbiota, with antimicrobial action against gram-positive bacteria, mainly *C. perfringens* and *S. aureus*.

ACKNOWLEDGMENTS

The authors acknowledge Joan Torrent (Oligo Basics Ind. Ltda., Cascavel, Paraná, Brazil) for providing the cashew nut shell oil and castor oil blend commercial.

REFERENCES

Abbas, R. Z., Z. Iqbal, A. Khan, Z. U. D. Sindhu, J. A. Khan, M. N. Khan, and A. Raza. 2012. Options for integrated strategies for the control of avian coccidiosis. Int. J. Agric. Biol. 14:1014–1020.

Abdel-Wareth, A. A. A., S. Kehraus, F. Hippenstiel, and K. M. Südekum. 2012. Effects of thyme and oregano on growth performance of broilers from 4 to 42 days of age and on microbial counts in crop, small intestine and caecum of 42-day-old broilers. Anim. Feed Sci. Technol. 178:198–202.

Apajalahti, J. H., L. K. Sarkilahti, B. R. Maki, J. P. Heikkinen, P. H. Nurminen, and W. E. Holben. 1998. Effective recovery of bacterial DNA and percent-guanine-plus-cytosine-based analysis of community structure in the gastrointestinal tract of broiler chickens. Appl. Environ. Microbiol. 64:4084–4088.

Baba, E., T. Ikemoto, T. Fukata, K. Sasai, A. Arakawa, and L. R. McDougald. 1997. Clostridial population and the intestinal lesions in chickens infected with *Clostridium perfringens* and *Eimeria necatrix*. Vet. Microbiol. 54:301–308.

Bess, F., A. Favero, S. L. Vieira, and J. Torrent. 2012. The effects of functional oils on broiler diets of varying energy levels. J. Appl. Poult. Res. 21:567–578.

Bortoluzzi, C., J. F. M. Menten, R. Pereira, N. S. Fagundes, G. S. Napty, A. A. Pedroso, A. D. Bigaton, and F. D. Andreote. 2015. Hops β-acids and zinc bacitracin affect the performance and intestinal microbiota of broilers challenged with Eimeria acervulina and Eimeria tenella. Anim. Feed Sci. Technol. 207:181–189.

Cao, G. T., X. F. Zeng, A. G. Chen, L. Zhou, L. Zhang, Y. P. Xiao, and C. M. Yang. 2013. Effects of a probiotic, Enterococcus faecium, on growth performance, intestinal morphology, immune response, and cecal microflora in broiler chickens challenged with Escherichia coli K88. Poult. Sci. 92:2949–2955.

Chapman, H. D., T. K. Jeffers, and R. B. Williams. 2010. Forty years of monensin for the control of coccidiosis in poultry. Poult. Sci. 89:1788–1801.

Collier, C. T., C. L. Hofacre, A. M. Payne, D. B. Anderson, P. Kaiser, R. I. Mackie, and H. R. Gaskins. 2008. Coccidia-induced mucogenesis promotes the onset of necrotic enteritis by supporting Clostridium perfringens growth. Vet. Immunol. Immunopathol. 122:104–115.

Cornelissen, J. B. W. J., W. J. C. Swinkels, W. A. Boersma, and J. M. J. Rebel. 2009. Host response to simultaneous infections with Eimeria acervulina, maxima and tenella: A cumulation of single responses. Vet. Parasitol. 162:58–66.

Cox, C. M., L. H. Sumners, S. Kim, A. P. McElroy, M. R. Bedford, and R. A. Dalloul. 2010. Immune responses to dietary beta-glucan in broiler chicks during an Eimeria challenge. Poult. Sci. 89:2597–2607.

Deplancke, B., and H. R. Gaskins. 2001. Microbial modulation of innate defense: Goblet cells and the intestinal mucus layer. Am. J. Clin. Nutr. 73:1131S–1141S.

DiAngelo, J. R., M. Bland, S. Bambina, S. Cherry, and M. Birnbaum. 2009. The immune response attenuates growth and nutrient storage in Drosophila by reducing insulin signaling. Proc. Natl. Acad. Sci. 106:20853–20858.

Ducatelle, R., V. Eeckhaut, F. Haesebrouck, and F. I. Van. 2015. A review on prebiotics and probiotics for the control of dysbiosis: present status and future perspectives. Animal 9:43–48.

Ferreira, A. J. P., and C. S. A. Ferreira. 2009. Estafilococose e Estreptococose aviária. Pages 475–482 in Doenças das aves. Junior A. Berchieri, E. N. Silva, J. Di Fabio, L. Sesti, and M. A. F. Zuanaze, 2th rev. ed. FACTA, Campinas.

Guban, J., D. R. Korver, G. E. Allison, and G. W. Tannock. 2006. Relationship of dietary antimicrobial drug administration with broiler performance, decreased population levels of lactobacillus salivarius, and reduced bile salt deconjugation in the ileum of broiler chickens. Poult. Sci. 85:2186–2194.

Guimarães, D. O., L. Da Silva Momesso, and M. T. Pupo. 2010. Antibióticos: Importância terapêutica e perspectivas para a descoberta e desenvolvimento de novos agentes. Quim. Nova 33:667–679.

Hume, M. E., S. Clemente-Hernández, and E. O. Oviedo-Rondón. 2006. Effects of feed additives and mixed Eimeria species infection on intestinal microbial ecology of broilers. Poult. Sci. 85:2106–2111.

Huyben, M. W., J. Sol, G. H. Counotte, M. P. Roumen, and J. Borst. 2001. Salinomycin poisoning in veal calves. Vet. Rec. 149:183–184.

Jonsson, P., and T. Wadstrom. 1993. Staphylococcus. Pages 21–35 in Pathogenesis of Bacterial Infections in Animals. 2nd ed. C. L. Gyles and C. O. Thoen, 1st ed. Lowa State University Press, Ames, IA.

Johnson, J., and W. M. Reid. 1970. Anticoccidial Drugs: Lesion scoring techniques in battery and floor-pen experiments with chickens. Exp. Parasitol. 28:30–36.

Kanehashi, S., R. Masuda, K. Yokoyama, T. Kanamoto, T. Nakashima, and T. Miyakoshi. 2015. Development of a cashew nut shell liquid (CNSL)-based polymer for antibacterial activity. J. Appl. Polym. Sci. 132:1–9.

Kense, M. J., and W. J. Landman. 2011. Enterococcus cecorum infections in broiler breeders and their offspring: molecular epidemiology. Avian Pathol. 40:603–612.

Kim, D. K., H. S. Lillehoj, S. H. Lee, S. I. Jang, M. S. Park, W. Min, E. P. Lillehoj, and D. Bravo. 2013. Immune effects of dietary anethole on Eimeria acervulina infection. Poult. Sci. 92:2625–2634.

Kim, G. B., Y. M. Seo, C. H. Kim, and I. K. Paik. 2011. Effect of dietary prebiotic supplementation on the performance, intestinal microflora, and immune response of broilers. Poult. Sci. 90:75–82.

Kley, M. A., E. O. Oviedo-Rondón, S. E. Dowd, M. Hume, and A. Nalian. 2012. Effect of Eimeria infection on cecal microbiome of broilers fed essential oils. Int. J. Poult. Sci. 11:747–755.

Klose, V., K. Bayer, R. Bruckbeck, G. Schatzmayr, and A. P. Loibner. 2010. *In vitro* antagonistic activities of animal intestinal strains against swine-associated pathogens. Vet. Microbiol. 144:515–521.

Kogut, M. H. 2013. The gut microbiota and host innate immunity: Regulators of host metabolism and metabolic diseases in poultry. J. Appl. Poult. Res. 22:637–646.

Laurent, F., R. Mancassola, S. Lacroix, R. Menezes, and M. Naciri. 2001. Analysis of chicken mucosal immune response to Eimeria tenella and Eimeria maxima infection by quantitative reverse transcription-PCR. Infect. Immun. 69:2527–2534.

Lunedo, R., M. F. Fernandez-Alarcona, F. M. S Carvalho, L. R. Furlan, and M. Macari. 2014. Analysis of the intestinal bacterial microbiota in maize- or sorghum-fed broiler chickens using real-time PCR. Br. Poult. Sci. 55:795–803. http://dx.doi.org/10.1080/00071668.2014.975781.

Mazzetto, S. L., D. Lomonaco, and G. Mele. 2009. Óleo da castanha de caju: oportunidades e desafios no contexto do desenvolvimento e sustentabilidade industrial. Quim. Nova 32:732–741.

M'Sadeq, S. A., S. B. Wu, R. A. Swick, and M. Choct. 2015. Dietary acylated starch improves performance and gut health in necrotic enteritis challenged broilers. Poult. Sci. 94:2434–2444.

Mohiti-Asli, M., and M. Ghanaatparast-Rash. 2015. Dietary oregano essential oil alleviates experimentally induced coccidiosis in broilers. Prev. Vet. Med. 120:195–202.

Murakami, A. E., C. Eyng, and J. Torrent. 2014. Effects of functional oils on coccidiosis and apparent metabolizable energy in broiler chickens. Asian Australas. J. Anim. Sci. 27:981–989.

Orengo, J., A. J. Buendía, M. R. Ruiz-Ibáñez, J. Madrid, L. Del Río, P. Catalá-Gregori, V. García, and F. Hernández. 2012. Evaluating the efficacy of cinnamaldehyde and Echinacea purpurea

Downloaded from https://academic.oup.com/ps/advance-article-abstract/doi/10.3382/ps/pez345/5524378 by Nottingham Trent University user on 01 July 2019

plant extract in broilers against Eimeria acervulina. Vet. Parasitol. 185:158–163.

Oviedo-Rondn, E. O., M. E. Hume, N. A. Barbosaa, N.K. Sakomura, G. Weber, and J. W. Wilsone. 2010. Ileal and caecal microbial populations in broilers given specific essential oil blends and probiotics in two consecutive grow-outs. Avian Biol. Res. 3:157–169.

Oviedo-Rondón, E. O., M. E. Hume, C. Hernández, and S. Clemente-Hernández. 2006. Intestinal microbial ecology of broilers vaccinated and challenged with mixed Eimeria species, and supplemented with essential oil blends. Poult. Sci. 85:854–860.

Pan, D., and Z. Yu. 2014. Intestinal microbiome of poultry and its interaction with host and diet. Gut Microbes 5:108–119.

Parasa, L. S., T. Sunita, and K. B. Rao. 2011. Acetone extract of Cashew (Anacardium occidentale, L.) nuts shelliquid against Methicillin resistant Staphylococcus aureus (MRSA) by minimum inhibitory concentration (MIC). J. Chem. Pharm. Res. 3:736–742.

Rinttilä, T., A. Kassinen, E. Malinen, L. Krogius, and A. Palva. 2004. Development of an extensive set of 16S rDNA-targeted primers for quantification of pathogenic and indigenous bacteria in faecal samples by real-time PCR. J. Appl. Microbiol. 97:1166–1177.

Rostagno, H. S., L. F. T. Albino, J. L. Donzele, P. C. Gomes, R. T. Oliveira, D. C. Lopes, A. S. Ferreira, L. S. T. Barreto, and R. F. Euclides. 2011. Brazilian tables for poultry and swine: Composition of feedstuffs and nutritional requirements. 3rd ed. UFV, Viçosa, Minas Gerais, Brazil.

SAS Institute. 2002. SAS/STAT User's Guide. Version 9. SAS Institute Inc., Cary, NC.

Sakomura, N. K., and H. S. Rostagno. 2016. Research methods in monogastric nutrition. 2. nd ed. FUNEP, Jaboticabal, São Paulo, Brazil.

Scheurer, W., P. Spring, and L. Maertens. 2013. Effect of 3 dietary phytogenic products on production performance and coccidiosis in challenged broiler chickens. J. Appl. Poult. Res. 22: 591–599.

Servin, A. L. 2004. Antagonistic activities of lactobacilli and bifidobacteria against microbial pathogens. FEMS Microbiol. Rev. 28:405–440.

Stanley, D., S. B. Wu, N. Rodgers, R. A. Swick, and R. J. Moore. 2014. Differential responses of cecal microbiota to fishmeal, Eimeria and Clostridium perfringens in a necrotic enteritis challenge model in chickens. PLoS One 9:e104739.

Stanley, V. G., C. Gray, M. Daley, W. F. Krueger, and A. E. Sefton. 2004. An alternative to antibiotic-based drugs in feed for enhancing performance of broilers grown on Eimeria Spp. Poult. Sci. 83:39–44.

Toyomizu, M., K. Okamoto, T. Ishibash, T. Nakatsu, and Y. Akiba. 2003. Reducing effect of dietary anacardic acid on body fat pads in rats. Ani. Sci. J. 74:499–504.

Vieira, C., S. Fetzer, and S. K. Sauer. 2001. Pro- and anti-inflammatory actions of ricinoleic acid: similarities and differences with capsaicin. Naunyn-Schmiedeberg's Arch. Pharmacol. 364:87–95.

Wise, M. G., and G. R. Siragusa. 2007. Quantitative analysis of the intestinal bacterial community in one- to three-week-old commercially reared broiler chickens fed conventional or antibiotic-free vegetable-based diets. J. Appl. Microbiol. 102: 1138–1149.

Yousaf, M. S., F. Goodarzi, W. Boroojeni, K. Vahjen, K. Männer, A. H. Hafeez, and J. Zentek. 2017. Encapsulated benzoic acid supplementation in broiler diets influences gut bacterial composition and activity. Br. Poult. Sci. 64:1–12.

Downloaded from https://academic.oup.com/ps/advance-article-abstract/doi/10.3382/ps/pez345/5524378 by Nottingham Trent University user on 01 July 2019
