# Supplementary material for: Modulation of the intestinal microbiota of broilers supplemented with monensin or functional oils in response to challenge by Eimeria spp
Source: PLoS One. 2020 Aug 7;15(8):e0237118. doi: 10.1371/journal.pone.0237118 (PMC7413546; doi:10.1371/journal.pone.0237118)
Supplement: S2 File — (PDF) [file pone.0237118.s006.pdf]

 © The Animal Consortium 2019
doi:10.1017/S1751731119000600

animal

Effect of functional oils on the immune response of broilers challenged with *Eimeria* spp.

P. O. Moraes[1†], I. Andretta[2], K. M. Cardinal[2], M. Ceron[2], L. Vilella[2], R. Borille[3], A. P. Frazzon[4], J. Frazzon[5], E. Santin[6] and A. M. L. Ribeiro[2]

[1]Department of Animal Science and Rural Development, Federal University of Santa Catarina, Florianópolis, Santa Catarina 88036-020, Brazil; [2]Department of Animal, Federal University of Rio Grande do Sul, Porto Alegre, Rio Grande do Sul 91540-000, Brazil; [3]Department of Animal Science and Biology Science, Federal University of Santa Maria, Palmeira das Missões, Rio Grande do Sul 98300-00, Brazil; [4]Department of Microbiology, Federal University of Rio Grande do Sul, Porto Alegre, Rio Grande do Sul 91540-000, Brazil; [5]Food Science and Technology Institute (ICTA), Federal University of Rio Grande do Sul, Porto Alegre, Rio Grande do Sul 91540-000, Brazil; [6]Department of Veterinary Medicine, Federal University of Paraná, Curitiba, Paraná 80035-050, Brazil

(Received 30 May 2018; Accepted 25 February 2019)

Infection with Eimeria *sp. results in the activation of multiple facets of the host immune system; the use of phytogenics can modulate the inflammatory response and improve the performance of the challenged animal. The aim of this study was to evaluate the effect of a commercial blend of cashew nut shell liquid (CNSL) and castor oil on the immune response of broilers challenged with coccidiosis. A total of 864 one-day-old male chicks (Cobb 500) were randomly distributed into six treatment groups (8 pens/treatment and 18 chicks/pen) in a three-by-two factorial design with three additives: control (non-additive), 100 ppm of monensin or 0.15% CNSL–castor oil. Challenge status was determined twice at 14 days of age. Unchallenged birds were inoculated by gavage with oocysts sporulated with* Eimeria tenella, Eimeria acervulina *and* Eimeria maxima. *Although the positive control (non-additive and challenged) and CNSL–castor oil treatment groups exhibited similar variation in weight gain (ΔBWG) compared to unchallenged birds fed without additives, the variation observed in birds fed diets containing CNSL–castor oil was associated with a higher maintenance requirement and not feed efficiency. In the second week after infection, ΔBWG of the CNSL–castor oil treatment group did not significantly change compared to the other treatment groups. At days 7 and 14 post-challenge, there was a higher excretion of oocysts in the control group, whereas the CNSL–castor oil and monensin groups did not differ. The CNSL–castor oil group exhibited increased gene expression of interferon (IFN), interleukin 6 (IL-6) and tumor necrosis factor (TNF), while the control group exhibited increased expression of cyclooxygenase (COX) and IL-1. The heterophils/lymphocyte ratio was low for the monensin treatment group. The unchallenged birds that received monensin treatment presented higher gene expression of IFN, COX and IL-1 compared to the other treatments, while the CNSL–castor oil group exhibited reduced gene expression, except for TNF. The commercial blend of cashew nut liquid and castor oil modulated the inflammatory response against* Eimeria *spp. In the absence of the parasite, there was no stimulation of genes involved in the inflammatory response, demonstrating that the blend is an effective tool in specifically modulating the immune system of birds afflicted with coccidiosis.*

Keywords: cashew nut, castor oil, challenge, coccidiosis, interleukins

Implications

Coccidiosis reduces broiler performance and increases the physiological cost of maintaining the immune system because of the effect of the parasite on intestinal health. Treatment with the blend of functional oils potentiated the host immunity against *Eimeria*, reducing the excretion of oocysts like ionophores; however, it reduced weight gain. Increased inflammatory response was essential for immune protection of the birds, albeit there was a smaller variation in weight gain after 2 weeks. These results demonstrated that the blend of functional oils mediated a slower recovery of the animals, but was as effective as treatment with ionophores.

Introduction

Coccidiosis usually occurs sub-clinically in birds, which makes it difficult to diagnose the disease in a timely manner and begin treatment before loss of performance occurs (Cornelissen *et al.*, 2009). Studies with the major species of *Eimeria* have revealed that innate and cell-mediated

† E-mail: priscila.moraes@ufsc.br

immunity have a fundamental action against the pathogen, both by the production of cytokines and by the direct cytotoxic attack of affected cells (Lillehoj and Choi, 1998; Laurent *et al.*, 2001). The primary proinflammatory cytokines, such as interleukin 1 (IL-1), IL-6 and tumor necrosis factor-α (TNF-α), are responsible for the acute-phase results that are associated to systemic and metabolic changes, including decreased appetite, increased basal metabolic rate, skeletal muscle degradation and acute-phase hepatic protein synthesis (Kogut and Klasing, 2009). Jiang *et al.* (2010) conducted lipopolysaccharide (LPS) challenge on animals and concluded that only 59% of performance loss was because of the reduction in feed intake, while 41% was because of other factors, likely associated with the immune response. Anticoccidial drugs are included in the animal diet as a preventative and continuous measure to minimize problems with coccidiosis. Monensin is an ionophore widely used in poultry production; however, many *Eimeria* spp. strains resistant to ionophores have been identified (Chapman *et al.*, 2010). Furthermore, the initiative to reduce the use of antibiotics as growth promoters has stimulated research to develop alternative methods to simultaneously reduce the impact of parasites and act as growth promoters.

Phytogenic additives may act as antimicrobial and anti-inflammatory agents with similar effects as drugs used in animal production, because generally, they are composed of a complex blend of volatile substances, such as terpene hydrocarbons, simple alcohols and aldehydes, among other pharmacologically active compounds (Applegate *et al.*, 2010). Among phytogenic additives, functional oils are defined as those that have an action beyond nutritional value (Murakami *et al.*, 2014). An example is the blend of cashew nut shell liquid and castor oil (CNSL–castor oil). Evaluating this blend in the diet of broilers challenged with coccidiosis, Murakami *et al.* (2014) observed increased weight gain and improved feed conversion, as well as a gain of 100 kcal of metabolizable energy in the diet compared to the control treatment group. According to Bess *et al.* (2012), this increase in energy availability may be associated with the antimicrobial and anti-inflammatory effects of functional oils. However, there are currently no *in vivo* studies that demonstrate the anti-inflammatory effect of these functional oils.

This study was developed to test the hypothesis that the commercial blend of cashew shell liquid and castor oil in the diet beneficially modulates the immune response of chickens challenged with coccidiosis.

Material and methods

Animals, diets and experimental design

A total of 864 one-day-old male chicks (Cobb 500) were obtained from a commercial hatchery and housed in two identical experimental rooms composed of 48 pens (8 pens/treatment and 18 birds/pens). The nutritional program consisted of three diets, pre-initial (days 1–7), initial (days 8–21) and growth (days 22–28), based on the levels of nutritional requirements recommended by the Brazilian Tables of Poultry and Swine (Rostagno *et al.*, 2011). The nutritional composition of each treatment group was the same, varying only the additive used in the diet. Metabolizable energy and crude protein in the three diets were 3000 kcal/kg and 22.2%, 3050 kcal/kg and 21% and 3150 kcal/kg and 19.69%, respectively.

The experimental design was completely randomized in a three-by-two factorial design with three additives: control (non-additive), 100 ppm of monensin or 0.15% CNSL–castor oil, to birds that were unchallenged or challenged with coccidiosis. Both additives, CNSL–castor oil and sodium monensin, were incorporated into the diets by replacing inert (kaolin) in the basal diet. We denoted the treatment group that was unchallenged and fed no additives as the negative control and the group that was challenged as the positive control.

Health challenge

At 14 days of age, sporulated oocysts of *Eimeria tenella* (10×10^3), *Eimeria acervulina* (200×10^3) and *Eimeria maxima* (80×10^3) were inoculated into challenged chickens by gavage. The oocysts were obtained from the *Laboratório de Biologia Molecular de Coccídias* (University of São Paulo, Brazil). Unchallenged chickens were inoculated with saline solution.

Sampling and data collection

Weight gain and feed intake were measured 7 and 14 days after challenging the health status of chickens when they were 21 and 28 days of age, respectively. We derived equations to study the relation between feed intake and weight gain. For this purpose, the performance responses of challenged birds in each treatment group were normalized to respective unchallenged control birds in each treatment group and expressed as a percentage of variation (Δ), according to the equation proposed by Pastorelli *et al.* (2012). Relative data were used to generate equations that estimated the relationship between the variation in weight gain (ΔG) and the variation in feed intake (ΔFI).

At 21 days of age (7 days post-challenge), three birds per experimental replicate were euthanized by cervical dislocation and samples (1 cm) of the duodenum (the final portion), jejunum (before the Meckel's diverticulum) and cecal tonsils were aseptically collected. Samples were washed in cold phosphate buffered saline, minced, immediately frozen in liquid nitrogen and stored at −80°C until gene expression analysis.

In addition, from these three birds, one was randomly selected to collect samples (5 cm) of the duodenum (the final portion), jejunum (before Meckel's diverticulum) and ileum (before cecum entry) to evaluate intestinal health. Before euthanasia, blood from the ulnar vein of one bird per experimental replicate was collected to perform a leukogram.

Oocyst count
At days 7 and 14 post-challenge, samples were collected from birds from five different entry points, forming a pool of samples per pen. The number of oocysts was determined as described by Costa and Paiva (2009), using a McMaster chamber and the formula: total oocysts/pen = (oocysts counted × dilution factor × [sample volume/counting chamber volume]/2).

Intestinal health index – 'I See Inside'
The 'I See Inside' (ISI) methodology (INPI BR 1020150036019) is an intestinal health index developed by Kraieski *et al.* (2016) that uses the following formula: ISI = Σ (EL × FI), where Σ represents the sum, EL is the lesion score (0–3, where 3 is the most severe) determined by observed histological changes, and FI is the pre-established Impact Factor (1–3, where 3 is the most severe) determined by the effect on organ function. Details of the ISI method are presented in Table 1.

Samples were fixed in buffered 10% formalin solution, and then prepared in paraffin blocks. Hematoxylin and eosin (HE) staining was conducted with Alcian Blue. Intestinal samples were evaluated by analyzing five villi per bird, using a Leica DM1000 LED optical microscope.

Gene expression
Total RNA was extracted from the pool of samples (20 mg) of duodenum, jejunum and cecal tonsils using the Invitrap® Spin Tissue RNA kit (Stratec, Berlin, DE), according to the manufacturer's instructions. RNA was eluted by washing the column membrane twice with RNase-free water (25 µL). The total concentration of RNA was determined by Qubit (Qubit 3.0 Fluorometer, Waltham, MA) and RNA purity was determined by measuring the ratio of optical density (OD) at 260 and 280 nm (NanoDrop-1000, Thermo Fisher Scientific, Waltham, MA, USA). Reverse transcription was performed using the high-throughput cDNA transcription kit QuantiTec Kit (Qiagen, Hilden, DE, Germany), following the manufacturer's protocol. cDNA was stored at −20°C. A Step One Plus (Applied Biosystems, Foster City, USA) was used for quantitative reverse transcription-polymerase chain reaction (qRT-PCR).

cDNA was diluted (1:100) in nuclease-free water and added to PCR mix (10 µL) containing 10 × PCR buffer (2.0 µL), of 50 mM $MgCl_2$ (1.6 µL), 10 µM of each primer (0.5 µL), 5 mM dNTP (0.2 µL), Syber green (2.0 µL) and Platinum® Taq DNA polymerase (0.05 µL). The conditions for qRT-PCR were 94°C for 5 min, 35 cycles at 94°C for 30 s, annealing temperature specific for each primer set (Table 2) for 30 s, and 72°C for 30 s. The specificity of PCR products was evaluated by analyzing dissociation curves. In addition, the size of amplicons was verified by gel electrophoresis. The values obtained for each gene were normalized and gene expression was calculated relative to the control group of unchallenged birds fed no additives, as described by Livak and Schmittgen (2001).

Table 1 *Intestinal health index classification table in broilers*

Alteration	Impact factor
Self-blade thickness	2
Epithelial thickness	1
Proliferation of enterocytes	1
Epithelial plasma infiltration	1
Mixed inflammatory infiltration of lamina propria	3
Goblets cells	2
Congestion	2
Necrosis (apical karyolysis)	3
Presence of oocysts	3
Maximum score[a]	54

[a]Maximum score represents the score obtained if all alterations were rated the maximum individual score of 3 (Adapted from Kraieski *et al.*, 2016).

Statistical analysis
Pearson's correlation coefficient was determined as a measure of the linear correlation between weight gain variation (ΔBWG) and feed intake variation (ΔFI) between the negative control group and individual treatment groups. The relationship between ΔFI and ΔBWG was analyzed using linear regression, where $\Delta BWG = \alpha + \beta \times \Delta FI$, as described by Pastrorelli *et al.* (2012). Analysis was conducted using the 'REG' function in SAS. The intercept (α) represents the reduction of BWG related to changes in maintenance requirements, independent of changes in FI. The slope (β) represents the extent of BWG change associated with the reduction of feed efficiency in challenged birds.

The factorial design was analyzed by ANOVA and included the effects of the additives, health challenge, and their interactions for all variables. The 'LSmeans' function was used to compare the means when statistically significant differences were calculated. The GLM procedure in SAS version 9.2 (SAS Institute, 2002) was used.

Results

Growth performance
We determined that the relationship between ΔBWG and ΔFI fit a linear regression (Figures 1 and 2). However, the ΔBWG was lower for all treatment groups in the second week compared to the first week, demonstrating that coccidiosis had the greatest impact within the first week of infection. In addition, the intercept values of the linear equations calculated from each treatment group at both time points were negative, suggesting that changes in maintenance requirements contributed to the reduction of ΔBWG, independent of variation in feed intake.

The ΔBWG observed in the treatment groups with challenged birds was due to decreased feed efficiency. However, the ΔBWG of the treatment group that was fed a diet with CNSL–castor oil was not affected by feed efficiency in the first week. In the CNSL–castor oil treatment group, the maintenance-related fraction was two-fold greater than that of the positive control group and 24-fold higher than the

Table 2 *Target genes and primers used for the analysis of mRNA expression in broilers*

Target	Sequence	ID	Reference
*β-actin_F	5′-ACCTGAGCGCAAGTACTCTGTCT 3'	NM_205518.1	Xie *et al.* (2014)
*β-actin_R	5′-CATCGTACTCCTGCTTGCTGAT-3′		
*GAPDH_F	5′-CCTAGGATACACAGAGGACCAGGTT-3′	NM_204305	Tan *et al.* (2014)
*GAPDH_R	5′-GGTGGAGGAATGGCTGTCA-3′		
IL-1B_F	5′-ACT GGG CAT CAA GGG CTA-3′	NM_204524	Tan *et al.* (2014)
IL-1B_R	5′-GGT AGA AGA TGA AGC GGG TC-3′		
IL-6_F	5′-TTTATGGAGAAGACCGTGAGG-3′	NM_204628	Long *et al.* (2011)
IL-6_R	5′-TGTGGCAGATTGGTAACAGAG-3′		
TNF-α_F	5′-TGCTGTTCTATGACCGCC-3′	AY765397	Hu *et al.* (2015)
TNF-α_R	5′-CTTTCAGAGCATCAACGCA-3′		
IFN-γ_F	5′-AGCTGACGGTGGACCTATTATT-3′	Y07922	Lee *et al.* (2012)
IFN-γ_R	5′-GGCTTTGCGCTGGATTC-3′		
COX-2_F	5′-GGTGAGACTCTGGAGAGGCAAC-3′	M64990	Laurent *et al.* (2001)
COX-2_R	5′-GTTGAACAGAAGCTCAGGGTCA-3′		
INOS_F	5′-CCTGTACTGAAGGTGGCTATTGG-3′	D85422	Cox *et al.* (2010)
INOS_R	5-AGGCCTGTGAGAGTGTGCAA-3′		
NF-κB_F	5′-GTGTGAAGAAACGGGAACTG-3′	NM_205129	Tan *et al.* (2014)
NF-κB_R	5′-GGCACGGTTGTCATAGATGG-3′		

F = forward; R = reverse; ID: GenBank access number; *Housekeeping genes.
GAPDH = glyceraldehyde 3-phosphate dehydrogenase; IL-1B = interleukin 1 beta; IL-6 = interleukin 6; TNF-α = tumor necrosis factor alpha; IFN-γ = interferon gamma; COX-2 = cyclooxygenase 2; INOS = nitric oxide synthase; NF-κB = nuclear factor kappa beta.

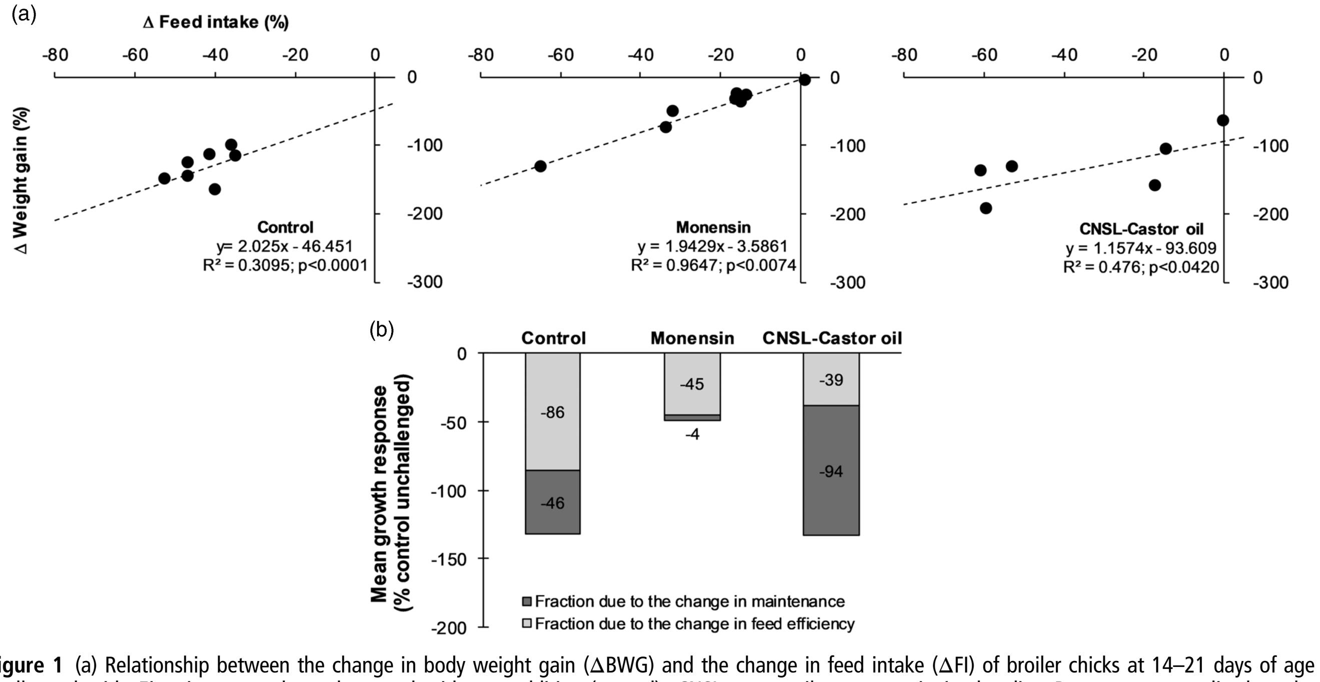

Figure 1 (a) Relationship between the change in body weight gain (ΔBWG) and the change in feed intake (ΔFI) of broiler chicks at 14–21 days of age challenged with *Eimeria* spp. and supplemented with non-additive (control), CNSL–castor oil or monensin in the diet. Data were normalized to the negative control group. (b) Partitioning of the reduction in ΔBWG into two factors: the change in maintenance (■ not associated with ΔFI) or the change in feed intake (□. associated with ΔFI). CNSL = cashew nut shell liquid.

monensin treatment group, which had the lowest ΔBWG. Groups fed diets containing CNSL–castor oil exhibited lower ΔBWG, resulting from changes in maintenance requirements (4%) and feed efficiency (11%), in the second week.

Oocyst count

The total oocyst counts of birds fed diets with monensin or CNSL–castor oil decreased in comparison to those of birds in the positive control group ($P < 0.05$) (Table 3). As expected, no oocysts were detected in unchallenged chickens.

Blood analysis and ISI score

We determined that the percentage of heterophils and the heterophil/lymphocyte ratio were affected by treatment with additives ($P < 0.05$). There were no statistically significant differences detected between the treatment groups of

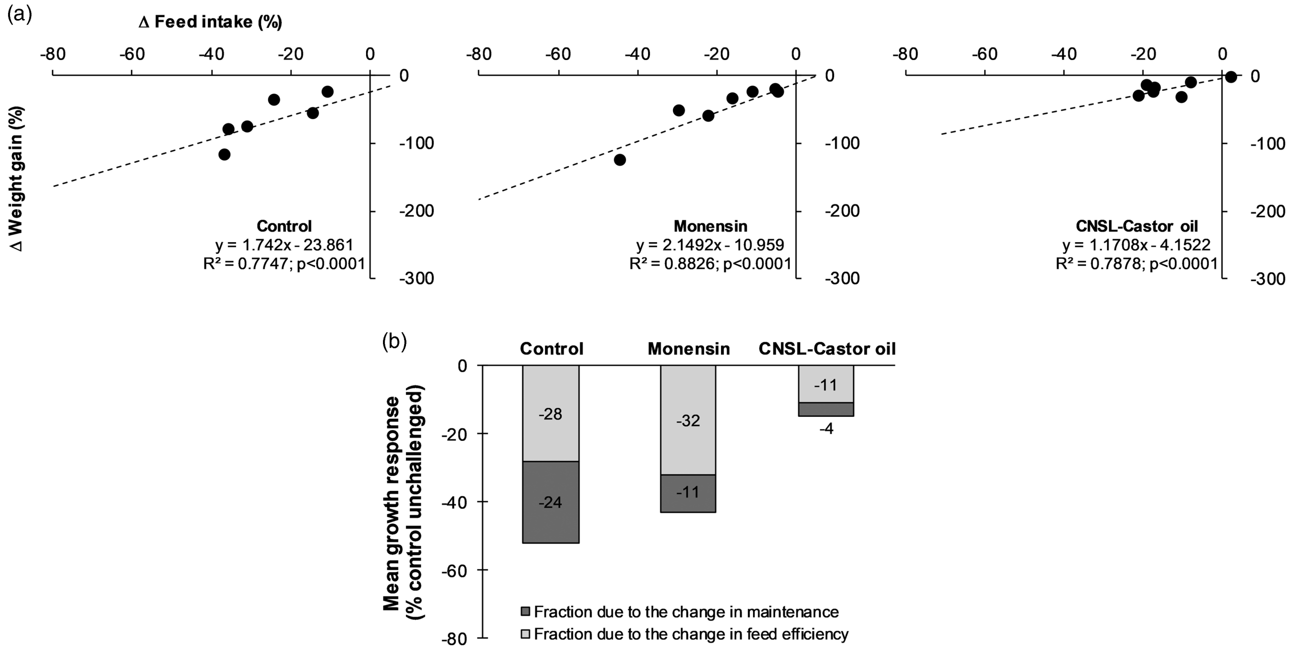

Figure 2 (a) Relationship between the change in body weight gain (ΔBWG) and the change in feed intake (ΔFI) of broiler chicks at 21–28 days of age challenged with *Eimeria* spp. and supplemented with non-additive (control), CNSL–castor oil or monensin (M) in the diet. Data were normalized to the negative control group. (b) Partitioning of the reduction in ΔBWG into two factors: the change in maintenance (■. not associated with ΔFI) or the change in feed intake (□. associated with ΔFI). CNSL = cashew nut shell liquid.

Table 3 *Excretion of oocysts in chickens challenged with coccidiosis at 7 and 14 days post-infection*

	Additives				
Days post-infection	Control	Monensin	CNSL–castor oil	*P*-value	SEM
7 days	2.11E + 05 a	8.44E + 04 b	7.51E + 04 b	3.97E-02	6.02 + 02
14 days	9.29E + 04 a	5.85E + 04 b	2.07E + 04 b	5.70E-03	2.19 + 02

CNSL = cashew nut shell liquid.
Statistical significance was denoted by different lowercase letters, according to the LSMEANS function in SAS ($P < 0.05$). $N = 8$ per treatment.

unchallenged birds ($P > 0.05$), whereas the positive control group of challenged birds and the CNSL–castor oil treatment group exhibited higher percentages of heterophils and heterophil/lymphocyte ratios compared to those of the monensin treatment group (Table 4). Moreover, both unchallenged and challenged birds receiving monensin had similar percentages of heterophils and heterophil/lymphocyte ratios. The change in total number of leukocytes and percentage of eosinophils between the treatment groups were not statistically significant. However, the percentages of lymphocytes and monocytes significantly differed between unchallenged and challenged birds ($P < 0.05$); unchallenged birds had higher percentages of lymphocytes and lower percentages of monocytes compared to challenged birds (data not shown).

Table 5 presents the mean scores obtained from the ISI morphometric index. Independent of whether the birds were unchallenged or challenged, CNSL–castor oil treatment resulted in higher ISI scores in treated birds, whereas monensin treatment did not affect ISI scores compared to the control group. The ISI scores are detailed in Supplementary Tables S1, S2 and S3.

Gene expression

mRNA expression of genes in samples of the duodenum and jejunum 7 days post-challenge (21 days of age) were quantified in Tables 6 and 7. mRNA expression of genes in samples of cecal tonsils did not exhibit any change. On the other hand, gene expression in samples of the duodenum and jejunum significantly increased in challenged birds ($P < 0.05$). In addition, mRNA expression of inducible nitric oxide synthase (INOS) was specifically affected by the additive fed to each treatment group ($P < 0.05$) (data not shown).

Challenged birds that received CNSL–castor oil in their diet exhibited lower INOS gene expression and higher nuclear factor kappa B (NF-κB) and IL-6 gene expression than birds receiving monensin ($P < 0.05$). Monensin treatment upregulated expression of interferon (IFN) and tumor necrosis factor (TNF) in the jejunum compared to the positive control group ($P < 0.05$). In the positive control group, the expression levels of IL-1 and cyclooxygenase (COX) were upregulated compared to levels in the monensin and CNSL–castor oil treatment groups. There were no statistically significant differences for either genes between the CNSL–castor oil and monensin

Table 4 *Percentage of heterophils and heterophil/lymphocyte ratio in broilers unchallenged or challenged with coccidiosis*

	Interaction on treatments									
	Unchallenged[1]			Challenged				*P*-value		
Variables	Cont	Mon	CN_CO	Cont	Mon	CN_CO	SEM	CH	Ad	CHxAd
Heterophils (%)	32.50^{b}	36.50^{b}	35.71^{b}	44.71^{a}	31.70^{b}	46.88^{a}	0.049	0.207	0.046	2.830
Heterophil/lymphobyte	0.65^{b}	0.75^{b}	0.83^{b}	1.40^{a}	0.72^{b}	1.60^{a}	0.002	0.042	0.429	0.134

[1]Challenged (CH) or unchallenged with pool *Eimeria* spp. at 14 days age of broilers.
Cont = Control without additives; Mon = Monensin 0.025 ppm; CN_CO = Commercial blend of cashew nut shell liquid and castor oil; Ad = Additives.
[a,b,c]Means with different superscript letters within a row represent significant differences ($P < 0.05$), 8 broilers per treatment.

Table 5 *Intestinal Health Index* of broilers at 21 days of age receiving different additives*

	Additives				*P*-value		
Gastrointestinal sections	Cont	Mon	CN_CO	SEM	CH[1]	Ad	CHxAd
Duodenum	14.55^{b}	14.12^{b}	17.75^{a}	1.345	<0.001	<0.001	0.598
Jejunum	18.77^{b}	16.74^{b}	23.23^{a}	1.387	<0.001	<0.001	0.481
Ileum	25.99^{b}	26.22^{b}	31.72^{a}	1.023	<0.001	<0.001	0.202

*Methodology 'I See Inside'.
Cont = Control without additives; Mon = Monensin; CN_CO = Commercial blend of cashew nut shell liquid and castor oil; Ad = Additives.
[a,b,c]Means with different superscript letters within a row represent significant differences ($P < 0.05$), 8 broilers per treatment.
[1]Challenged (CH) or unchallenged with pool *Eimeria* spp. at 14 days age of broilers.

Table 6 *mRNA expression of various interleukin genes in the duodenum of broilers challenged (CH) or unchallenged (Un) with coccidiosis*

	Unchallenged			Challenged				*P*-value		
Genes expression	Cont	Mon	CN_CO	Cont	Mon	CN_CO	SE	CH	Ad	CHxAd
INOS	1.000^{Bb}	1.019^{Ba}	0.972^{Bc}	1.009^{Ab}	1.029^{Aa}	1.008^{Ab}	0.0077	< 0.0001	< 0.0003	< 0.0001
TNF-α	1.000^{Bb}	1.029^{Aa}	1.026^{Ba}	1.031^{Aa}	1.027^{Aa}	1.036^{Aa}	0.0063	< 0.0001	< 0.0001	< 0.0001
IFN-γ	1.000^{Bb}	1.031^{Aa}	0.988^{Bc}	1.034^{Aa}	1.032^{Aa}	1.038^{Aa}	0.0066	< 0.0001	< 0.0001	< 0.0001
NF-κB	1.000^{Bb}	1.029^{Ba}	1.022^{Ba}	1.037^{Ab}	1.039^{Ab}	1.045^{Aa}	0.0060	< 0.0001	< 0.0003	< 0.0001
COX-2	1.000^{Bb}	1.021^{Aa}	0.977^{Bc}	1.028^{Aa}	1.032^{Aa}	1.024^{Aa}	0.0098	< 0.0001	< 0.0012	< 0.0001
IL-1B	1.000^{Bb}	1.020^{Aa}	1.006^{Bb}	1.033^{Aa}	1.028^{Ab}	1.024^{Ab}	0.0059	0.0069	0.0008	< 0.0001
IL-6	1.000^{Aa}	0.997^{Aa}	0.983^{Bb}	0.991^{Ab}	0.992^{Ab}	1.021^{Aa}	0.0089	0.1082	< 0.0001	0.0131

Gene expression was calculated relative to the negative control group. Data were presented as log 10.
Cont = Control without additives (Ad); Mon = Monensin; CN_CO = commercial blend of cashew nut shell liquid and castor oil; INOS = nitric oxide synthase; TNF-α = tumor necrosis factor alpha; IFN-γ = interferon gamma; NF-κB = nuclear factor kappa beta; COX-2 = cyclooxygenase 2; IL-1B = interleukin 1 beta; IL-6 = interleukin 6.
[A,B,C]Means with different superscript letters within a row represent significant differences for challenge status.
[a,b,c]Means with different superscript letters within a row represent significant differences for additive used ($P < 0.05$).

treatment groups ($P > 0.05$). Challenged birds that did not receive CNSL–castor oil exhibited lower expression of the genes evaluated in this study compared to the other treatment groups ($P < 0.05$), except for TNF expression, which did not change in the jejunum ($P > 0.05$) but decreased in the duodenum ($P < 0.05$). Treatment with monensin increased expression of IFN, COX and IL-1 to levels higher than those measured in the other treatment groups ($P < 0.01$).

Discussion

The CNSL–castor oil additive stimulated the inflammatory response of birds by mediating an immune response against the parasite within a week after infection. Our results demonstrated that CNSL–castor oil treatment increased gene expression of proinflammatory interleukins, such as IFN, TNF and IL-6, heterophil/lymphocyte ratio in the blood, ISI scores and maintenance expenditure compared to the control group. However, birds treated with CNSL–castor oil excreted the same number of oocysts as birds treated with monensin. In the positive control group, challenged birds receiving no additives in the diet exhibited decreased inflammatory response and increased excretion of oocysts, showing that the parasite proliferated more successfully in the absence of treatment.

Overall, as expected, birds challenged with coccidiosis had more severe ISI scores, increased heterophil/lymphocyte ratio

Table 7 *mRNA expression of various interleukin genes in the jejunum of broilers challenged (CH) or unchallenged (Un) with coccidiosis*

	Unchallenged			Challenged				*P*-value		
Genes expression	Cont	Mon	CN_CO	Cont	Mon	CN_CO	SE	CH	Ad	CHxAd
INOS	1.000^{Bc}	1.006^{Bb}	1.014^{Aa}	1.023^{Aa}	1.024^{Aa}	1.018^{Ab}	0.005	0.0459	0.1739	< 0.0001
TNF-α	1.000^{Bc}	1.021^{Bb}	1.054^{Aa}	1.012^{Aa}	0.099^{Aa}	1.012^{Aa}	0.008	0.0057	0.0026	0.0054
IFN-γ	1.000^{Bb}	1.009^{Ba}	0.982^{Bc}	1.018^{Aa}	1.012^{Aa}	0.998^{Ab}	0.005	< 0.0001	< 0.0001	0.0008
NF-κB	1.000^{Ba}	1.010^{Ba}	0.960^{Bb}	1.033^{Aa}	1.015^{Ab}	1.030^{Aa}	0.006	< 0.0001	< 0.0001	< 0.0001
COX-2	1.000^{Bb}	1.005^{Ba}	0.998^{Bb}	1.031^{Ab}	1.033^{Aa}	1.008^{Aa}	0.006	0.0006	< 0.0001	< 0.0002
IL-1B	1.000^{Bc}	1.020^{Aa}	1.011^{Bb}	1.022^{Aa}	1.010^{Bb}	1.015^{Bb}	0.005	0.0157	0.0726	< 0.0001
IL-6	1.000^{Ba}	0.985^{Ab}	0.999^{Ba}	1.014^{Ab}	0.989^{Ac}	1.023^{Aa}	0.006	0.3674	< 0.0001	< 0.0001

Gene expression was calculated relative to the negative control group. Data were presented as log 10.
Cont = Control without additives (Ad); Mon = Monensin; CN_CO = commercial blend of cashew nut shell liquid and castor oil; INOS = nitric oxide synthase; TNF-α = tumor necrosis factor alpha; IFN-γ = interferon gamma; NF-κB = nuclear factor kappa beta; COX-2 = cyclooxygenase 2; IL-1B = interleukin 1 beta; IL-6 = interleukin 6
^{A,B,C}Means with different superscript letters within a row represent significant differences for challenge status.
^{a,b,c}Means with different superscript letters within a row represent significant differences for additive used ($P < 0.05$).

and increased expression of INOS, TNF, IFN, NF-κB, COX, IL-1 and IL-6 within the first week of infection, according to analysis of intestinal samples, which was characteristic of an ongoing inflammatory response against the pathogen (Laurent *et al.*, 2001; Cox *et al.*, 2010; Zhou *et al.*, 2014; Chen *et al.*, 2016; Zhang *et al.*, 2016). According to Klasing (2004) and Kogut and Klasing (2009), the acute phase response is the first defense mechanism of the immune response that facilitates systemic and metabolic alterations.

The immune response against the coccidiosis parasite is complex and many associated mechanisms are not fully understood. Both the innate and cell-mediated immune system have a fundamental action in response to a pathogen. The immune system does not prevent invasion of sporozoites into enterocytes, rather it inhibits their development (Allen and Fetterer, 2002). Innate immunity recognizes a pathogen through toll-like receptors that use the myeloid differentiation primary response 88 (MYD88) adapter protein to activate gene expression of four primary pro-inflammatory proteins, IL-1, IL-6, TNF-α and IFN-γ (Tizard, 2009). These cytokines play important regulatory roles modulating the course of the immune response during infection (Allen and Fetterer, 2002). We demonstrated that CNSL–castor oil modulated the immune system by increasing gene expression of TNF-α, IL-6 and IFN-γ and reducing expression of IL-1 and COX-2, compared to the positive control group. The efficacy of the CNSL–castor oil additive was observed by the reduction of oocysts in birds in the treatment group.

CNSL–castor oil treatment effectively reduced excretion of oocysts by increasing expression of IFN-γ. Upregulated IFN-γ expression stimulates cell-mediated immunity, which has been reported to be crucial in the immune response against *Eimeria* (Lillehoj and Choi, 1998). IFN-γ protein expressed mainly by $CD4^+$ cells can directly inhibit sporozoite development by increasing the cytotoxic activity of $CD8^+$ cells and activating macrophages (Allen and Fetterer, 2002). Lee *et al.* (2008) demonstrated that the use of a phytogenic additive composed of *Prunus salicina* in animal feed increased expression of IFN-γ, mediating protective immunity against coccidiosis, indicated by a reduction in oocyst excretion and weight loss 10 days after infection. The results obtained in this study presented similar conclusions to this previous report.

TNF-α and IL-6 are inflammatory response markers stimulated by macrophages and NF-κB (Kim *et al.*, 2008), and in the present study, the heterophil/lymphocyte ratio increased in challenged birds, reflecting the severity of ISI scores in challenged birds. According to Kraieski *et al.* (2016), increased ISI scores are associated with increased infiltration of lymphocytes in the lamina propria, epithelial thickness, goblet cells, congestion and enterocyte proliferation or infiltration of inflammatory cells.

Our results demonstrated that there was a relationship between feed intake and weight gain, especially in terms of the partitioning of the observed variations. For example, although the ΔBWG was similar (–132%) in the positive control group and CNSL–castor oil treatment group, the partitioning of the ΔBWG differed between the groups. In the positive control group, the ΔBWG was more significantly attributed to changes associated with feed efficiency (–86%), such as a reduction in feed consumption, whereas the ΔBWG in the CNSL–castor oil treatment group was more likely caused by changes associated with maintenance and not ΔFI (–94%). Zhang *et al.* (2016) reported that the ΔBWG in broilers challenged with coccidiosis was attributed to changes in maintenance requirements. The nutrient requirements for maintenance of birds were reported to increase when challenged with coccidiosis within the first week of infection (Laurent *et al.*, 2001; Cornelissen *et al.*, 2009; Cox *et al.*, 2010). Several reported maintenance requirements include increased metabolic costs for repairing damaged tissues, immune system stimuli and reduced capacity to utilize and convert nutrients into energy (Chen *et al.*, 2016; Grenier *et al.*, 2016). The innate immune system may cause additional collateral damage to the body, such as fever and inflammatory reactions, that consumes resources, reducing nutrient reserves and increasing catabolism (Klasing and Iseri, 2013; Iseri and Klasing, 2014). Owing to these costly consequences, inflammation is a highly undesirable phenomenon in animal

production. However, the increased inflammatory response observed within a week of infection in challenged birds treated with CNSL–castor oil additive was necessary to transform the immune system to effectively fight against coccidiosis and other pathogenic bacteria to prevent intestinal dysbiosis.

In fact, 2 weeks after infection, the CNSL–castor oil treatment group exhibited reduced populations of *Clostridium perfringens* and *Staphylococcus aureus*, which are opportunistic and potentially harmful bacteria (Moraes *et al.*, 2017). Therefore, we hypothesized that the mechanism of action of CNSL–castor oil additive was like a vaccine, potentiating the host immune system against additional pathogens while simultaneously modulating the target pathogen. The monensin treatment group exhibited decreased inflammatory response, characterized by downregulated expression of TNF, IFN, NF-κB, COX, IL-1 and IL-6, in at least one of the intestinal samples analyzed per bird. This may be because of the fact that monensin was a more effective anticoccidial additive, targeting the parasite when it was outside the host cells in the form of merozoites, before any inflammatory response could peak (Laurent *et al.*, 2001; Chapman *et al.*, 2010; Cox *et al.*, 2010).

In unchallenged birds, CNSL–castor oil treatment decreased gene expression of most of the ILs, whereas monensin treatment increased expression of IFN-γ, COX-2 and IL-1. This may have resulted because of the more balanced microbiota of birds in the CNSL–castor oil treatment group. After analysis of microbiota of birds at 28 days of age, we confirmed that there was a larger population of *Lactobacillus* spp. in the CNSL–castor oil treatment group (Moraes *et al.*, unpublished). These bacteria can modulate the gene expression of cytokines, toll-like receptors and T cells (Brisbin *et al.*, 2011).

The commercial blend of CNSL and castor oil modulated the inflammatory response of birds challenged with coccidiosis against *Eimeria* spp. In the absence of the parasite, inclusion of the additive in the diet did not unnecessarily stimulate expression of genes involved in the inflammatory response, demonstrating that the blend was an effective tool in modulating the immune system with high specificity.

Acknowledgments

The authors acknowledge Joan Torrent (Oligo Basics) for providing the cashew nut shell oil and castor oil blend commercial. Part of this work was previously presented at the 106th Poultry Science Association Annual Meeting conference 2017.

Declaration of interest

The authors declare that there is no conflict of interest.

Ethics statement

The Institutional Animal Care and Use Committee at Federal University of Rio Grande do Sul reviewed and approved the protocol used in the present study (register number 29814).

Software and data repository resources

The authors declare that data are not deposited in an official repository.

Supplementary material

To view supplementary material for this article, please visit https://doi.org/10.1017/S1751731119000600

References

Allen PC and Fetterer R 2002. Recent advances in biology and immunobiology of Eimeria species and in diagnosis and control of infection with these coccidian parasites of poultry. Clinical Microbiology Reviews 15, 58–65.

Applegate T, Klose V, Steiner T, Ganner A and Schatzmayr G 2010. Probiotics and phytogenics for poultry: Myth or reality? Journal of Applied Poultry Research 19, 194–210.

Bess F, Favero A, Vieira SL and Torrent J 2012. The effects of functional oils on broiler diets of varying energy levels. The Journal of Applied Poultry Research 21, 567–578.

Brisbin JT, Gong J, Orouji S, Esufali J, Mallick AI, Parvizi P, Shewen PE and Sharif S 2011. Oral treatment of chickens with lactobacilli influences elicitation of immune responses. Clinical and Vaccine Immunology 18, 1447–1455.

Chapman HD, Jeffers TK and Williams RB 2010. Forty years of monensin for the control of coccidiosis in poultry. Poultry Science 89, 1788–1801.

Chen X, Zhang Q and Applegate TJ 2016. Impact of dietary branched chain amino acids concentration on broiler chicks during aflatoxicosis. Poultry Science 95, 1281–1289.

Cornelissen JB, Swinkels WJ, Boersma WA and Rebel JM 2009. Host response to simultaneous infections with Eimeria acervulina, maxima and tenella: a cumulation of single responses. Veterinary Parasitology l 162, 58–66.

Costa CAF and Paiva DP 2009. Cultivo in vivo, in vitro e diagnóstico específico de Eimeria spp. de Gallus Gallus, 1st edition. Embrapa Informação Tecnológica, Brasília, DF, Brasil.

Cox CM, Sumners LH, Kim S, McElroy AP, Bedford MR and Dalloul RA 2010. Immune responses to dietary beta-glucan in broiler chicks during an Eimeria challenge. Poultry Science 89, 2597–2607.

Grenier B, Dohnal I, Shanmugasundaram R, Eicher SD, Selvaraj RK, Schatzmayr G and Applegate TJ 2016. Susceptibility of broiler chickens to coccidiosis when fed subclinical doses of deoxynivalenol and fumonisins-special emphasis on the immunological response and the mycotoxin interaction. Toxins 8, 231–253.

Hu JL, Yu H, Kulkarni RR, Sharif S, Xie MY and Gong J 2015. Modulation of cytokine gene expression by selected Lactobacillus isolates in the ileum, caecal tonsils and spleen of Salmonella-challenged broilers. Avian Pathology 44, 463–469.

Iseri V and Klasing K 2014. Changes in the amount of lysine in protective proteins and immune cells after a systemic response to dead *Escherichia coli*: implications for the nutritional costs of immunity. Integrative and Comparative Biology 54, 922–930.

Jiang Z, Schatzmayr G, Mohnl M and Applegate TJ 2010. Net effect of an acute phase response—partial alleviation with probiotic supplementation. Poultry Science 89, 28–33.

Kim J-Y, Park SJ, Yun K-J, Cho Y-W, Park H-J and Lee K-T 2008. Isoliquiritigenin isolated from the roots of Glycyrrhiza uralensis inhibits LPS-induced iNOS and COX-2 expression via the attenuation of NF-κB in RAW 264.7 macrophages. European Journal of Pharmacology 584, 175–184.

Klasing KC 2004. The costs of immunity. Acta Zoologica Sinica 50, 961–969.

Klasing K and Iseri V 2013. Recent advances in understanding the interactions between nutrients and immunity in farm animals. In Proceedings of the 4th Energy and Protein Metabolism and Nutrition in Sustainable Animal Production, 9–12 September 2013, California, USA, pp. 353–359.

Kogut MH and Klasing K 2009. An immunologist's perspective on nutrition, immunity, and infectious diseases: introduction and overview. Journal of Applied Poultry Research 18, 103–110.

Kraieski AL, Hayashi RM, Sanches A, Almeida GC and Santin E 2016. Effect of aflatoxin experimental ingestion and Eimeria vaccine challenges on intestinal histopathology and immune cellular dynamic of broilers: applying an Intestinal Health Index. Poultry Science 96, 1078–1087.

Laurent F, Mancassola R, Lacroix S, Menezes R and Naciri M 2001. Analysis of chicken mucosal immune response to *Eimeria tenella* and *Eimeria maxima* infection by quantitative reverse transcription-PCR. Infection and Immunity 69, 2527–2534.

Lee SH, Lillehoj HS, Lillehoj EP, Cho SM, Park DW, Hong YH, Chun HK and Park HJ 2008. Immunomodulatory properties of dietary plum on coccidiosis. Comparative Immunology, Microbiology and Infectious Diseases 31, 389–402.

Lee KW, Lillehoj HS, Lee SH, Jang SI, Park MS, Bautista DA, and Lillehoj EP 2012. Effect of dietary antimicrobials on immune status in broiler chickens. Asian-Australasian Journal of Animal Sciences, 25, 392–392.

Lillehoj H and Choi K 1998. Recombinant chicken interferon-gamma-mediated inhibition of *Eimeria tenella* development in vitro and reduction of oocyst production and body weight loss following *Eimeria acervulina* challenge infection. Avian Diseases 307–314.

Livak KJ and Schmittgen TD 2001. Analysis of relative gene expression data using real-time quantitative PCR and the 2(-Delta Delta C(T)) Method. Methods 25, 402–408.

Long FY, Guo YM, Wang Z, Liu D, Zhang BK and Yang X 2011. Conjugated linoleic acids alleviate infectious bursal disease virus-induced immunosuppression in broiler chickens. Poultry Science, 90, 1926–1933.

Moraes P, Gouvêa F, Schroeder B, Villela L, Torrent J and Ribeiro A 2017. Effect of a commercial blend of cashew nut shell liquid and castor oil on the microbiota of broilers challenged with Eimeria spp. In Proceedings of the 106th Poultry Science Association Annual Meeting Conference, 17–20 July 2017, Orlando Florida, pp. 60.

Murakami AE, Eyng C and Torrent J 2014. Effects of functional oils on coccidiosis and apparent metabolizable energy in broiler chickens. Asian-Australasian Journal of Animal Sciences 27, 981–989.

Pastorelli H, van Milgen J, Lovatto P and Montagne L 2012. Meta-analysis of feed intake and growth responses of growing pigs after a sanitary challenge. Animal 6, 952–961.

Rostagno H, Ferreira A, Barreto S and Euclides S R 2011. Tabelas brasileiras para aves e suínos, 4th revised edition. Universidade Federal de Viçosa, Viçosa, MG, Brasil.

Tan J, Applegate TJ, Liu S, Guo Y and Eicher SD 2014. Supplemental dietary L-arginine attenuates intestinal mucosal disruption during a coccidial vaccine challenge in broiler chickens. British Journal of Nutrition 112, 1098–1109.

SAS 2002. Proprietary software release 9.2. SAS Institute, Cary, NC, USA.

Tizard I 2009. Imunologia Veterinária, 9th revised edition. Elsevier, Rio de Janeiro, RJ, Brazil.

Xie J, Tian C, Zhu Y, Zhang L, Lu L and Luo 2014. Effects of inorganic and organic manganese supplementation on gonadotropin-releasing hormone-I and follicle-stimulating hormone expression and reproductive performance of broiler breeder hens. Poultry Science 93, 959–969.

Zhang Q, Chen X, Eicher SD, Ajuwon KM and Applegate TJ 2016. Effect of threonine deficiency on intestinal integrity and immune response to feed withdrawal combined with coccidial vaccine challenge in broiler chicks. British Journal of Nutrition 116, 2030–2043.

Zhou Z-y, Hu S-j, Wang Z-y, Guo Z-l, Qin B and Nie K 2014. Expression of chicken toll-like receptors and signal adaptors in spleen and cecum of young chickens infected with *Eimeria tenella*. Journal of Integrative Agriculture 13, 904–910.
